# Succinate Dehydrogenase-Regulated Phosphoenolpyruvate Carboxykinase Sustains Copulation Fitness in Aging *C. elegans* Males

**DOI:** 10.1016/j.isci.2020.100990

**Published:** 2020-03-19

**Authors:** Jimmy Goncalves, Yufeng Wan, Xiaoyan Guo, Kyoungsun Rha, Brigitte LeBoeuf, Liusuo Zhang, Kerolayne Estler, L. René Garcia

**Affiliations:** 1 Department of Biology, Texas A&M University, College Station, TX 77843, USA; 2 Institute for Neurodegenerative Diseases, University of California, San Francisco, CA 94158, USA; 3 Institute of Oceanology, Chinese Academy of Sciences, Qingdao, Shandong 266071, China

**Keywords:** Biological Sciences, Animal Physiology, Behavior Genetics

## Abstract

Dysregulated metabolism accelerates reduced decision-making and locomotor ability during aging. To identify mechanisms for delaying behavioral decline, we investigated how *C. elegans* males sustain their copulatory behavior during early to mid-adulthood. We found that in mid-aged males, gluco-/glyceroneogenesis, promoted by phosphoenolpyruvate carboxykinase (PEPCK), sustains competitive reproductive behavior. *C. elegans'* PEPCK paralogs, *pck-1* and *pck-2,* increase in expression during the first 2 days of adulthood. Insufficient PEPCK expression correlates with reduced *egl-2*-encoded *ether-a-go-go* K+ channel expression and premature hyper-excitability of copulatory circuits. For copulation, *pck-1* is required in neurons, whereas *pck-2* is required in the epidermis. However, PCK-2 is more essential, because we found that epidermal PCK-2 likely supplements the copulation circuitry with fuel. We identified the subunit A of succinate dehydrogenase SDHA-1 as a potent modulator of PEPCK expression. We postulate that during mid-adulthood, reduction in mitochondrial physiology signals the upregulation of cytosolic PEPCK to sustain the male's energy demands.

## Highlights

• *C. elegans* upregulates *pck-1-* and *pck-2*-encoded PEPCK during early adulthood
• Loss of PEPCK causes premature male copulatory behavior decline
• Epidermal PEPCK is required to sustain the copulatory fitness
• Subunit A of succinate dehydrogenase antagonizes PEPCK expression

## Graphical Abstract

**Figure:**
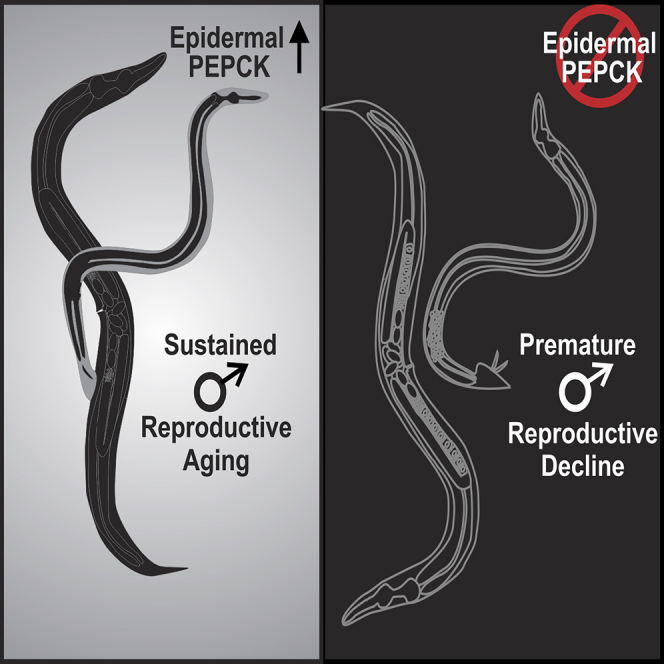

## Introduction

Although reduction in decision-making and locomotor ability is an inevitable consequence of aging, many studies have suggested that nutrition and its effects on metabolism will modify the rate and manifestation of behavioral decline (Canevelli et al., 2016, Garland et al., 2016, Norton et al., 2012, Sanders et al., 2016, Smith and Blumenthal, 2016). Dysregulated cellular and mitochondrial metabolism, which can occur during an animal's lifespan, has been demonstrated to reduce viability and synaptic and contractile functions of neurons and muscles (Baraibar et al., 2016, Bender et al., 2006, Camandola and Mattson, 2017, Kraytsberg et al., 2006, Saxton and Sabatini, 2017). The dysregulated metabolism can disrupt behavior by limiting ATP production or indirectly by generating excessive reactive by-products, such as reactive oxygen species and/or advanced glycation end products, which impair cellular and mitochondrial functions. Progressive accumulated damage can limit the regeneration of metabolic intermediates for sustaining neuronal and muscular performance (Allaman et al., 2015, Trushina and McMurray, 2007).

For adult males of the *Caenorhabditis elegans* laboratory N2 strain, reproductive-specific behaviors decline faster than general behaviors shared with hermaphrodites. When reared in groups, the average lifespan of well-fed adult males is 6–8 days, but their lifespan can be extended up to 15 days, if they are reared in solitude (Gems and Riddle, 2000, Shi et al., 2017). However, despite differences in lifespan and rearing conditions, copulatory behavior decays after 2 days of adulthood. The age-related decline in copulation efficiency is due to increases in muscle excitability, which lead to motor coordination defects during mating attempts (Guo et al., 2012).

The N2 male's competitive copulation efficiency, during their first 2 days of adulthood, is affected by its nutritional status. For example, males that are transiently starved between the end of their last larval molt and up to the first 18 h of adulthood, but then refed, have (1) their copulation ability extended by an extra day (Guo et al., 2012) and (2) have enhanced meiotic activity of their germ cells (Chou et al., 2019). If the males continue to be starved, their copulation ability is attenuated (Honjoh et al., 2017). The hormesis response of transient starvation stress promotes calcium calmodulin kinase II and *ether-a-go-go*-like K+ channel functions. These molecules mitigate the increases of age-related muscle excitability (Guo et al., 2012, LeBoeuf et al., 2011). In contrast to starved animals, if well-fed males are mutant for the NAD-dependent histone deacetylase gene *sir-2.1,* their copulation ability prematurely declines between days 1 and 2. The behavioral defects of young *sir-2.1* mutant males resemble older wild-type males. Other general behaviors of the *sir-2.1* mutant males, such as locomotion and feeding, do not differ from wild-type (Guo and García, 2014).

The *sir-2.1*-encoded NAD-dependent protein deacetylase monitors cellular NAD+ levels to regulate the expression of genes involved in metabolism and stress handling in *C. elegans* (Berdichevsky et al., 2006, Mouchiroud et al., 2013, Viswanathan et al., 2005, Wang et al., 2006). In *sir-2.1* mutant adult males, catabolic genes involved in glycolysis and the tricarboxylic acid (TCA) cycle are upregulated, but reactive oxygen species (ROS) scavenger genes are down-regulated. The misregulated energy consumption in the *sir-2.1* mutants abnormally increases muscle excitability and compromises the coordination of different sex-specific muscle contractions during the copulation step of male spicule penetration and sperm transfer. Other prematurely upregulated genes in *sir-2.1* mutants are the anabolic genes *pck-1* and *pck-2* (Guo and García, 2014), implicated in gluconeogenesis and glyceroneogenesis (Yuan et al., 2012, Yuan et al., 2016). A possibility arises that in the *sir-2.1* mutant, the upregulation of these anabolic genes is a compensation response to consequences from abnormal upregulation of catabolic genes. Consistent with this idea, the mating defect of day 1 *sir-2.1* mutant males is aggravated if they also contain a loss-of-function mutation in either *pck-1* or *pck-2*. In wild-type males, *pck-1* and *pck-2* RNA levels naturally increase between days 1 and 2. This observation suggests that the role these molecules play in reducing the consequences of *sir-2.1* deficiency in day 1 males might also occur to sustain mating fitness for wild-type day 2 males (Guo and García, 2014).

*pck-1* and *pck-2* both encode the enzyme phosphoenolpyruvate carboxykinase (PEPCK) (Yuan et al., 2012, Yuan et al., 2016). PEPCK phosphorylates and decarboxylates oxaloacetate (OAA), a TCA cycle intermediate, to form phosphoenolpyruvate (PEP). In mammalian cells, there are cytoplasmic and mitochondrial forms of the enzyme (Ballard and Hanson, 1967, Nordlie and Lardy, 1963). Depending on the cell's immediate needs, PEP can be used for gluco-/glyceroneogenesis or to re-form pyruvate (Chakravarty et al., 2005). Consequently, the pyruvate can re-enter the TCA cycle, undergo a transamination reaction with glutamate to produce alanine and α-ketoglutarate or be reduced to lactate. In mammalian cells, if glucose levels get too high, cytoplasmic PEPCK can also be acetylated by the p300 acetyltransferase to cause the enzyme to catalyze the reverse reaction of generating OAA from PEP, presumably to feed the TCA cycle and promote further energy production. Mammalian SIRT1 NAD-dependent protein deacetylase has also been shown to deacetylate PEPCK to maintain the canonical cataplerotic function of generating PEP from OAA (Latorre-Muro et al., 2018).

Groups have reported that increased cytoplasmic PEPCK levels correlate with sustained or enhanced animal muscle function and longevity. For example, in rodents, artificial overexpression of cytoplasmic PEPCK in skeletal muscle increases behavioral activity, mitochondrial content, and the usage of fatty acids (Hakimi et al., 2007). Similarly, artificial overexpression of cytoplasmic PEPCK increases *C. elegans*' lifespan, whereas elimination of cytoplasmic PEPCK reduces the worm's longevity. In *C. elegans* body wall muscles, cytoplasmic PEPCK levels increase during normal aging and even more so during caloric restriction-induced lifespan extension (Yuan et al., 2012, Yuan et al., 2016). In regard to *C. elegans* male mating behavior, the natural upregulation of *pck-1* and *pck-2* might enhance cellular function by either providing or shunting OAA to or from the TCA cycle, offsetting aging or *sir-2.1* mutation-induced metabolic dysfunction.

In this work we investigated PEPCK's metabolic contributions to the maintenance of N2 male's copulation fitness. We examined why males upregulate their two cytoplasmic PEPCK-expressing genes during the first 2 days of adulthood and how *pck-1* and *pck-2* differentially promote copulation activity from different tissues. We also used forward genetics to identify metabolic factors that contribute to the upregulation of PEPCK in both males and hermaphrodites, and we found that loss-of-function mutations in the mitochondrial succinate dehydrogenase subunit A of the electron transport chain complex II increase pck*-1* and *pck-2* expression. The succinate dehydrogenase complex participates in both the TCA cycle and the electron transport chain. We found that pharmacological perturbations of other electron transport chain components do not increase cytoplasmic PEPCK levels to levels similar to reducing complex II function, suggesting that *C. elegans* monitors the mitochondrial TCA cycle to adjust the abundance of PEPCK.

## Results

### Expression of PCK-1 and PCK-2 in *C. elegans* Hermaphrodites and Males

*C. elegans pck-1*, *pck-2,* and *pck-3* have sequence homology to PEPCK genes of other animal species. The amino acid sequences for PCK-1 and PCK-2 show 71% identity with each other and 58% identity to mammalian PEPCK orthologs (Figures S1 and S2). Both retain the conserved catalytic amino acids required for PEP formation, supporting their functionality (Figure S1) (Carlson and Holyoak, 2009, Sullivan and Holyoak, 2007). In contrast, PCK-3 has much lower similarity (23%–25% identity) to PCK-1 and PCK-2 and lacks some of the conserved catalytic amino acids (Figure S3). Yuan and colleagues showed that PCK-1 and PCK-2 account for all measurable OAA to PEP catalysis; cell extracts from *pck-1(0); pck-2(0)* double mutants lack detectable PEPCK activity, signifying that *pck-3* might not function as a PEPCK paralog (Yuan et al., 2016). Immunological staining of PCK-1, determined by antibodies to rat PEPCK (Ballard and Hanson, 1969), suggested that PCK-1 is expressed in the cytoplasm of worm muscles, intestine, and to a lesser extent, the pharynx (Yuan et al., 2012, Yuan et al., 2016); the expression of PCK-2 was not reported. However, due to the high amino acid identity, cross-reactivity of PCK1 and PCK2 to the rat antibody might complicate the expression profiles of the two paralogs. Therefore we studied the localization and expression of PCK-1 and PCK-2 by tagging a fluorescent protein to each gene.

**Figure 1:**
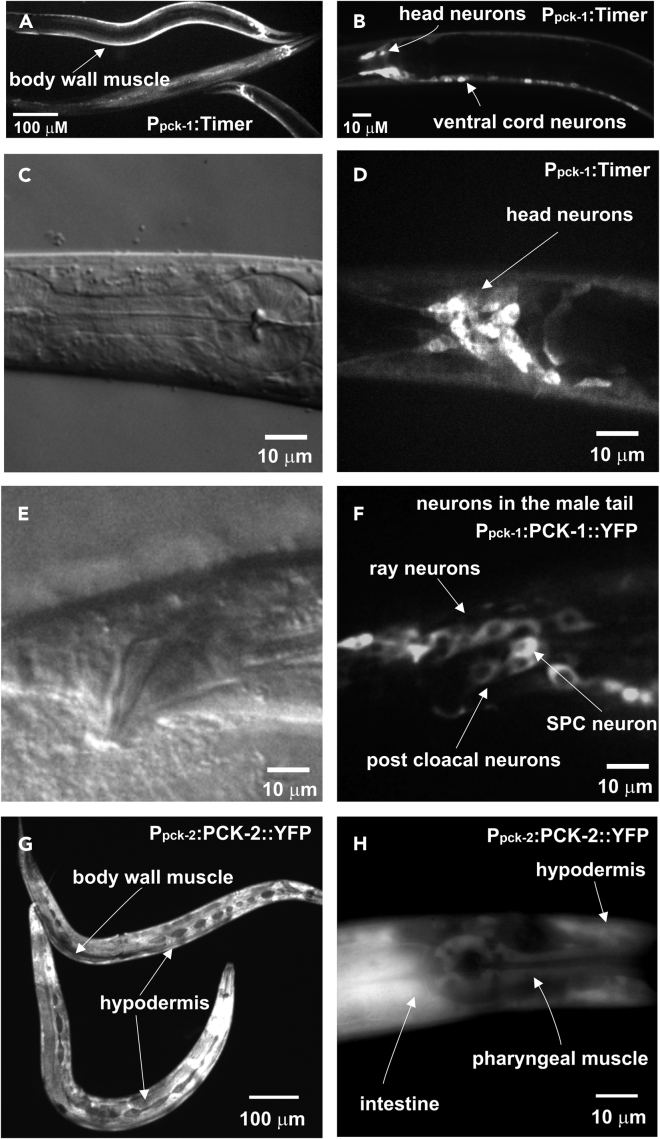
PCK-1 and PCK-2 Expression in *C. elegans* (A) Transgene *pck-1* promoter driving Timer protein fluorescence in the body wall muscle of 24-h adult hermaphrodites. Head neurons can also be seen in these animals. (B) Transgene *pck-1* promoter driving Timer protein fluorescence in ventral cord neurons of an L4 hermaphrodite. (C) Differential interference contrast (DIC) image of the head region of the L4 hermaphrodite from (B). (D) Transgene *pck-1* promoter driving Timer protein fluorescence in head neurons of the L4 hermaphrodite from (C). (E) DIC image of a 24-h adult male tail. (F) Transgene *pck-1* promoter driving *pck-1* genomic DNA-YFP fusion in neurons, including the male-specific ray and post-cloacal and spicule neurons of the male from (E). (G) CRISPR/Cas9-generated, genomic-expressed PCK-2::YFP expressed in the body wall muscles and epidermis of 24-h adult hermaphrodites. (H) CRISPR/Cas9-generated genomic-expressed PCK-2::YFP expressed in the pharyngeal muscles, epidermis, and intestine of a 24-h adult hermaphrodite. This figure is related to Figures S1–S5.

To explore further the published *pck-1* expression, we characterized yellow fluorescence protein (YFP) or Timer expression from transgenes that contained the 2.8-kb *pck-1* promoter. YFP was fused downstream of just the promoter, to the promoter and a mini-*pck-1* gene containing the first three introns, or to the promoter and intronless *pck-1* cDNA. The three different constructs were made to test if *pck-1* intronic and/or exonic sequences might contribute to the expression pattern. Consistent with the previous report on the hermaphrodites' expression, we confirmed here that for both sexes, all three of *pck-1*-expressing reporters express in head and body wall muscles (Figure 1A). However, we found that the reporters also express in ventral cord and preanal neurons (Figure 1B) and ∼20 nerve ring neurons per side in the head (Figures 1C and 1D). In the tail region, both larval males and larval/adult hermaphrodites express the transgenes in the dorsal rectal ganglion and three neurons per side in the tail. The adult male shows additional expression in sex-specific neurons of the ray, postcloacal, and spicule sensilla (Figures 1E and 1F). None of the constructs promote expression in male or hermaphrodite sex muscles, intestine, or pharyngeal muscles. The expression pattern suggests that PCK-1 contributes to the functional metabolism of *C. elegans* neurons and the muscles they innervate.

Previous work suggested that PCK-1 functions in the cytoplasm, whereas its paralog PCK-2 functions in the mitochondria (Yuan et al., 2016). YFP fluorescence from the *pck-1*::YFP cDNA fusion is found in the cytosol, confirming that PCK-1 is a cytoplasmic enzyme (Figures 1A–1F). Aside from large gene expression studies (Hunt-Newbury et al., 2007), no comprehensive expression pattern for PCK-2 has been published. To address this issue, we explored where PCK-2 is located in *C. elegans*, both with respect to tissue and subcellular specificity. The sequence of the first 19 amino acids of PCK-2's N terminus differs from that of PCK-1; however, sequence predictions suggest that the N terminus of PCK-2 does not resemble a standard mitochondrial targeting sequence (TargetP1.1 Server: www.cbs.dtu.dk Emanuelsson et al., 2000; MitoFates: mitf.cbrc.jp Fukasawa et al., 2015; MITOPROT: https://ihg.gsf.de/ihg/mitoprot.html Claros and Vincens, 1996). To determine the tissue and subcellular localization of PCK-2, we used CRISPR/Cas9 to delete and replace the stop codon of genomic *pck-2* with YFP coding sequence, generating endogenously tagged *pck-2*::YFP (Figure S4A). Males and hermaphrodites that contain the CRISPR/Cas9-generated *pck-2*::YFP fusion allele (*pck-2(rg551)*) are superficially wild-type in behavior and development. We found that PCK-2::YFP accumulated in the intestine, epidermis, body wall muscle, pharyngeal muscles, and sex muscles of both sexes (Figures 1G and 1H); no neural expression was detectable. Subcellular analysis with a mitochondrial marker showed that PCK-2 has no association with mitochondria, but resides in the cytoplasm (Figure S5). Expression of PCK-2 in the intestine and pharyngeal muscle is consistent with the earlier report that these tissues contain PEPCK activity (Yuan et al., 2016); however, in these tissues, the expression pattern suggests that the enzyme is likely expressed from *pck-2,* instead of the *pck-1*. Taken together with previously published data, PCK-1 and PCK-2 are likely cytosolic PEPCK paralogs that are expressed in different tissues, but express redundantly in the body wall muscles.

### PCK-2 Acts with PCK-1 to Sustain Male Copulation Fitness during First 2 Days of Adulthood

**Figure 2:**
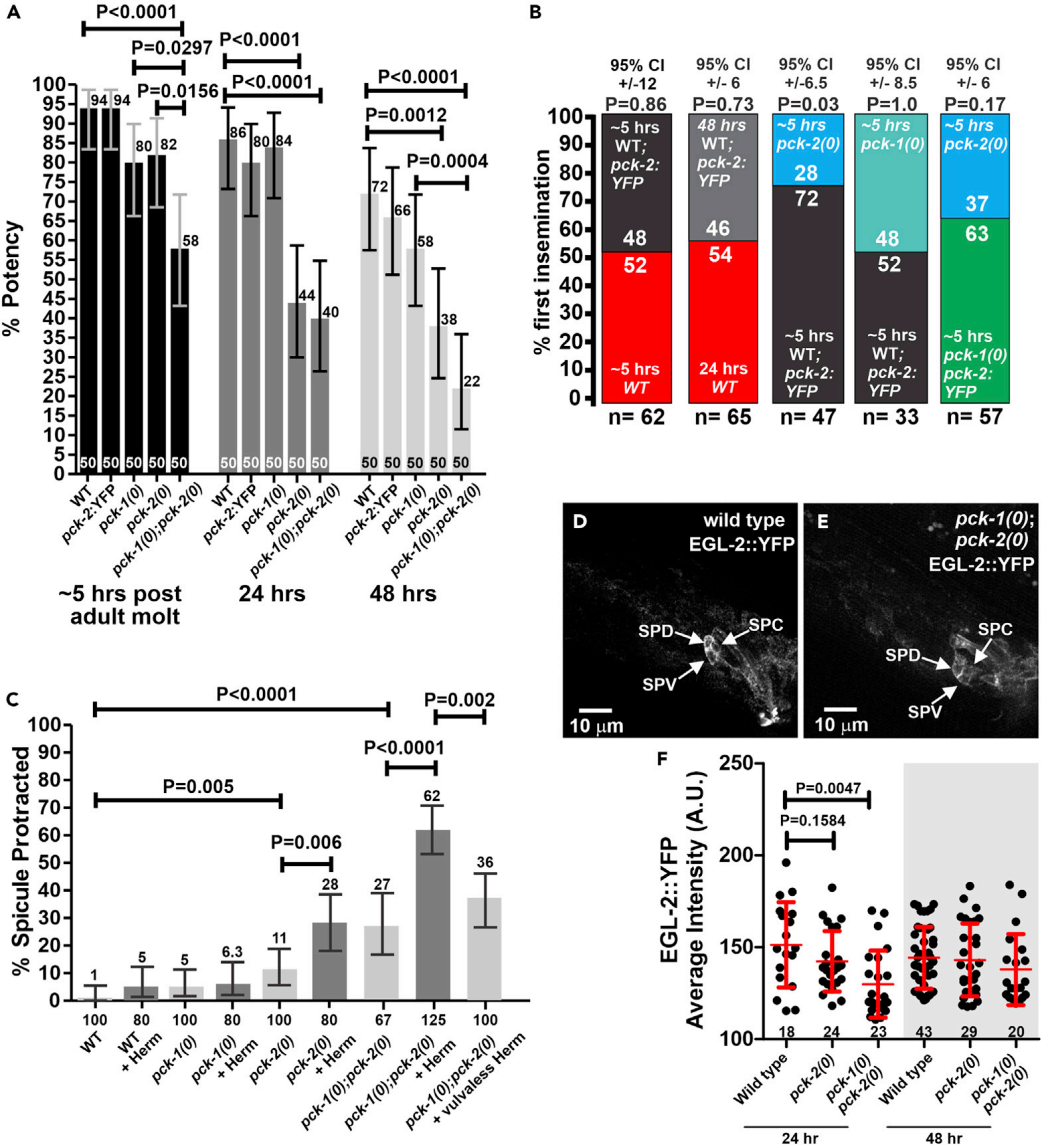
Male Mating Potency and Copulation Fitness (A) Mating potency of wild-type and PEPCK mutant males determined at the newly molted adult stage and 24 and 48 h of adulthood. Single aged males were paired with single 24-h *pha-1*(ts) hermaphrodites for 5 days. Numbers of males assayed for each age group are listed at the bottom of the bars. The % potency values are listed at the top of the bars. p values were determined using Fisher's exact test. (B) Copulation competition; n = number of independent trials. The numbers within the colored bars are the percent victory trials for the specific genotype. 95% CI (confidence interval); p values were determined using Fisher's exact test. (C) Percentage of 24-h-old males with constitutively protracted spicules. Groups of 20–25 males were incubated with or without five 24-h-old wild-type hermaphrodites (Herm); vulvaless hermaphrodites contain the mutation *let-23(sy1)*. Numbers of males assayed are listed at the bottom of the bars. The % spicule protracted values are listed at the top of the bars. p values were determined using Fisher's exact test. (A–C) Error bars represent the 95% confidence intervals. (D and E) Confocal images of EGL-2::YFP expression in one set of the bilateral SPD, SPV, and SPC spicule neurons; in the images, anterior region of the male tail is to the left and dorsal is to the top. Panel D depicts the tail of a wild-type male. Panel E depicts the tail of a *pck-1(0)*;*pck-2(0)* double mutant male. (F) Average fluorescence intensity of a region of interest (ROI) encompassing the SPD, SPV, and SPC neurons on one side of a male. Fluorescence values are in arbitrary units (A.U). Bars and whiskers represent mean and standard deviation. Numbers of males quantified are listed at the bottom of the graph. Each dot represents an ROI of a single male. p value was determined using the Mann-Whitney test. This figure is related to Figure S4.

The expression of PCK-2::YFP suggests that this paralog of PEPCK is cytoplasmic and functions in the epidermis, all muscles, and in the intestine. However, before characterizing the properties of the fusion protein further, we tested if the YFP tag on PCK-2 disrupts the enzyme's function or worm's behavior. Previously, we reported that at 20°C, the ability for well-fed *C. elegans* N2 males to sire at least one progeny (the copulation potency assay) with 24-h adult hermaphrodites declines after 48 h of adulthood (Figure 2A). When *pck-2* function is disrupted via the deletion allele *(ok2586),* referred to as *pck-2(0),* copulation decline is accelerated; the mutant's ability to sire progeny decreases after 24 h of adulthood (Guo et al., 2012). The accelerated decline is likely due to decrease of behavioral fitness rather than sperm quality, because reduced potency is not observed if *pck-2(0)* males copulate with older and easier-to-mate hermaphrodites (48 h of adulthood); 80% potent for 24-h *pck-2(0)* males, n = 50 and 84% for 24-h wild-type males, n = 50. If the CRISPR/Cas9 knock-in YFP tag disrupts the genomic PCK-2's function in copulation, then aging *pck-2*::YFP males should display a reduced copulation potency similar to the *pck-2(0)* mutants. However, in the first 2 days of adulthood, males expressing PCK-2::YFP sired progeny with an efficiency similar to wild-type (Figure 2A). Thus the YFP tag on *pck-2*-expressed PEPCK does not obviously accelerate male impotence. In contrast to the *pck-2* mutation, the *pck-1* deletion allele *(ok2098),* referred to as *pck-1(0),* does not affect copulation kinetics of mutant males up till 48 h of adulthood (Figure 2A) (Guo and García, 2014). Although PCK-1 appears to be non-essential, the *pck-1*-encoded PEPCK is likely used for mating, because we observed that *pck-1(0)*; *pck-2(0)* double mutant males display synthetically lower copulation efficiency right after L4 molt (∼5 h) (Figure 2A).

The potency assay indicated that during early adulthood (∼5 h after molt), the *pck-1(0)* and *pck-2(0)* single mutants are similar to wild-type, suggesting that the two PEPCK enzymes might compensate for each other. However, the compensation during this temporal window might not be equivalent. The potency assay addresses if wild-type and mutant males are competent to sire at least one progeny; however, it does not test if aging factors or genetic compensation affect competitive fitness. To determine fitness, we conducted a mating competition assay. In this assay, we paired two males (one containing a fluorescent marker) with one *fog-2* mutant female on a 5-mm bacterial lawn and observed which male transfers sperm first; fluorescence status of the cross-progeny was used to validate the paternity. Results from the competition assay provided further insight into the differential requirement of PEPCK (Figure 2B). When ∼5-h adult PCK-2::YFP and non-tagged wild-type males compete, 50% of the time either male transfers sperm first, reaffirming that the YFP tag does not interfere with mating behavior. The YFP tag allowed us to ask if copulation fitness is reduced in the first 48 h of adulthood. Surprisingly, the competition results between 24-h PCK-2 non-tagged and 48-h PCK-2::YFP wild-type adult males indicate that the differentially aged males have equivalent fitness. In contrast, although young adult *pck-1(0)* and *pck-2(0)* single mutants show similar mating ability in the potency assay (Figure 2A), *pck-2(0)* mutants are less fit than *pck-1(0)* mutants. When ∼5-h adult *pck-1(0)* or *pck-2(0)* single mutants compete against ∼5-h adult PCK-2::YFP wild-type males, the *pck-1(0)* males display similar fitness to the wild-type, but the *pck-2(0)* single males show reduced fitness. However, *pck-1(0)* males might not be entirely wild-type in behavior. When *pck-1(0)* and *pck-2(0*) males competed against each other, *pck-1(0)* males generally won, but unlike the competition between wild-type and *pck-2(0)* males, the margin was too low to be statistically different (Figure 2B). These data suggest that compared with *pck-1*, PEPCK expressed from *pck-2* provides a larger contribution to the competitive fitness of early and mid-aged N2 male mating behavior.

Even though the copulation fitness was lower, casual mating observations of *pck-2(0)* single and *pck-1(0)*; *pck-2(0)* double mutants did not identify any steps in copulation that were grossly defective; however, after 24 h, a proportion of *pck-2(0)* single and *pck-1(0)*; *pck-2(0)* double mutant males displayed spontaneous irreversible spicule protractor muscle spasm (Figure 2C). The constitutively contracted protractor muscles cause the copulatory spicules to dangle out the cloaca, rendering the male impotent. This *pr*otraction *c*onstitutive phenotype (Prc) can be seen in old wild-type males (older than 3 days) and in mutants that are defective in regulating membrane excitability of the spicules' sex-specific neurons and muscles (Garcia and Sternberg, 2003, Jobson et al., 2015). Interestingly, we observed that extended copulation attempts with mating-reticent young hermaphrodites increased the frequency of the Prc phenotype in *pck-2(0)* single- and even more so in *pck-1(0)*; *pck-2(0)* double mutants (Figure 2C). When we replaced those hermaphrodites with mutants that do not develop a functional vulva, the frequency of the Prc phenotype decreased (Figure 2C). These observations indicate that vulva insertion attempts exacerbate the *pck-1(0)*; *pck-2(0)*-induced defect. Taken together, the synthetic *pck-1(0)*; *pck-2(0)* phenotype suggests that for the *pck-2(0)* single mutant, PCK-1 provides partial compensatory PEPCK function up to the first 24 h of adulthood, but for the *pck-1(0)* single mutant, PCK-2 can provide compensatory PEPCK function at least up till the first 48 h of adulthood that we tested; the role for the two PEPCK likely involves sustaining differential regulation of neuron and muscle excitability in the copulation circuitry during adulthood.

In earlier reports, we observed the EGL-2 *ether-a-go-go*-like K+ channel expression in sex muscles increases during the first 48 h of male adulthood to regulate membrane excitability of copulation cellular components (LeBoeuf et al., 2011). Similar to the *pck-2(0)* phenotype, deletion of EGL-2 also results in 24–48 h premature decline of male mating potency (Guo et al., 2012). The studies indicated that the voltage-gated K+ channel is required to modulate male sex muscles' membrane potential during aging and food stress; however, in those studies, neural expression from extrachromosomal EGL-2 transgenes was not consistent. To circumvent the expression variabilities with extrachromosomal transgenes, we used CRISPR/Cas9 to knock-in YFP into the terminus of the genome-encoded *egl-2* gene. In the *egl-2*::YFP genomic recombinants, we found that in addition to male sex muscles, EGL-2::YFP is also expressed on the cell bodies, neural processes, and sensory endings of the male ray, post-cloacal, and spicule sensory neurons (Figure S4C).

To determine if the K+ channel's expression in the sensory and motor neurons and protractor muscles used for spicule insertion behavior underlies the copulation defects, we crossed the genomic *egl-2*::YFP knock-in allele into *pck-2(0)* and *pck-1(0)*; *pck-2(0)* mutants. We quantified EGL-2::YFP expression in the male's protractor muscles and SPC, SPD, and SPV neurons (Figures S4D and 2D–2F). The spicule protractor muscles control the movement of the spicules. The SPC neurons are presumptive proprioceptive sensory motor neurons that sense the position of the spicules during copulation attempts and subsequently stimulate tonic contraction of the spicule protractor muscles to extend the spicules through the vulval opening. The SPD and SPV sensory neurons send their sensory processes through the shaft of the spicules, where their ciliated endings are exposed at the tips of the spicules. These sensory neurons presumably sense the hermaphrodite's uterine environment and subsequently coordinate sperm transfer with the duration of spicule penetration (LeBoeuf et al., 2014). Previous work showed that the expression of a transcriptional *egl-*2 promoter reporter construct increased in wild-type sex muscles between 24 and 48 h of adulthood (LeBoeuf et al., 2011). Here we observed that the protractor muscles' EGL-2::YFP levels also increased between 24 and 48 h in wild-type males (Figure S4D). However, aging did not significantly change the K+ channel levels in the three neurons we quantified (Figure 2F). In contrast to the wild-type and the *pck-2(0)* single mutant, at 24 h of adulthood, the EGL-2::YFP fluorescence in the muscles, as well as the three sex neurons, was lower in the *pck-1(0)*; *pck-2(0)* mutant, consistent with their behavioral defect. However, as the animals aged by 48 h, the K+ channels eventually increased to wild-type levels (Figures 2D–2F). These results suggest that both PCK-1 and PCK-2 function promotes EGL-2 levels in the copulatory circuit early within the first 24 h of adulthood.

### PCK-1 Functions in Cholinergic Neurons and PCK-2 in Muscle and Epidermis to Promote Mating Success

**Figure 3:**
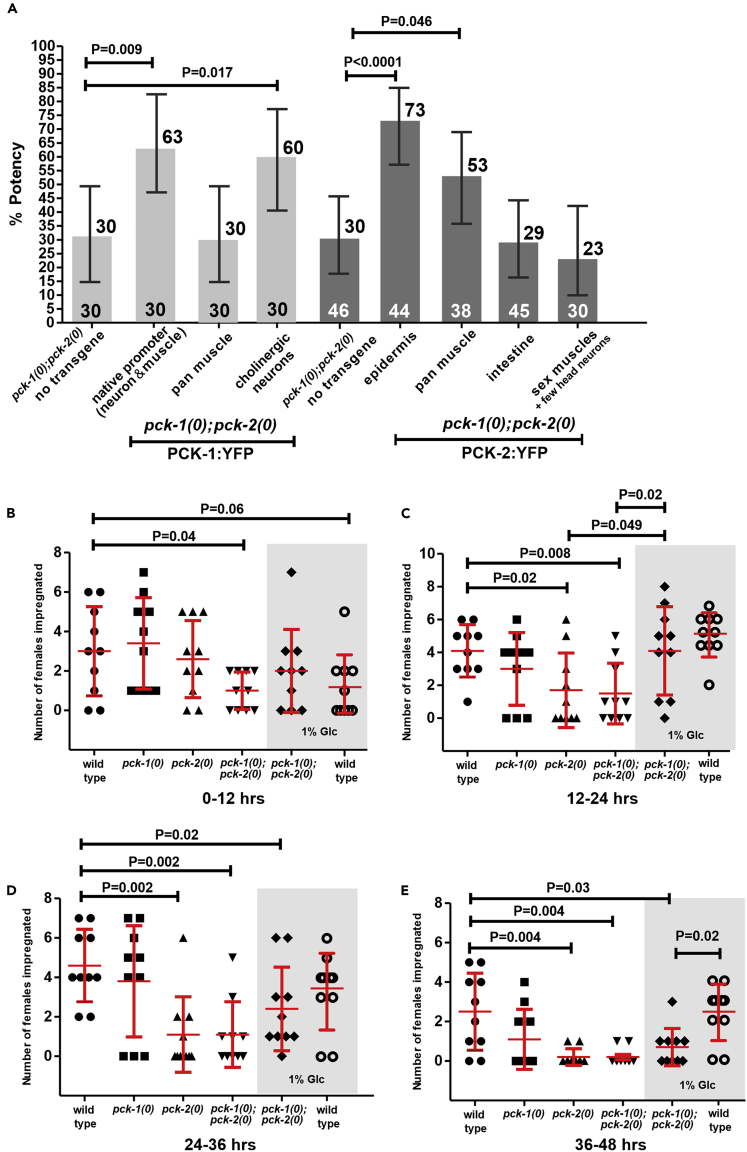
Male Mating Potency and Efficiency Rescue in PEPCK-Deficient Mutants (A) Mating potency of 24-h *pck-1(0)*; *pck-2(0)* adult males expressing transgenic tissue-specific-expressed *pck-1*::YFP or *pck-2*::YFP. Numbers of males assayed are listed at the bottom, and % potency values are listed at the top of the bars. p Values were determined using Fisher's exact test. Error bars represent the 95% confidence intervals. (B–E) Copulation endurance assay. x axis depicts genotype and growth condition. y axis depicts the number of females that were impregnated. The data for each condition constitutes 10 trials with one male per -trial (see also Figure S6). Each symbol represents a single male. The gray window denotes matings wherein animals were incubated with 1% glucose. Bars and whiskers represent mean and standard deviation. p values were determined using the Mann-Whitney test. (B) Number of females impregnated in the first 12 h after adult molt. (C) Number of females impregnated between 12 and 24 h. (D) Number of females impregnated between 24 and 36 h. (E) Number of females impregnated between 36 and 48 h. This figure is related to Figure S6.

To understand how PCK-1 and PCK-2 impact mating behavior, we needed to determine where they were functioning. To address this question, we tested which tissue(s) require *pck-1* and *pck-2* to restore mating potency to 24-h adult *pck-1(0)*; *pck-2(0)* double mutant males. We rationalized that restoring the different PEPCK gene to their relevant tissues should make the double mutant male behave similar to a PEPCK single mutant. The PEPCK double mutant displays a more severe defect than *pck-1(0)* or *pck-2(0)* alone, allowing for easier determination of tissue-specific function. Plasmids containing a *pck-1* cDNA or a mini *pck-2* gene (where the large second intron was removed) expressed by the *pck-1* promoter, pan-muscle promoter (*unc-103A* promoter), the acetylcholine vesicular transporter promoter (*unc-17* promoter), an epidermis promoter (*dpy-7* promoter), intestinal promoter (*gtl-1* promoter), or a sex muscle promoter (*unc-103E* promoter) were injected into the double mutant. The mating potencies of transgenic males indicated that expressing *pck-1* in cholinergic neurons and expressing *pck-2* in either epidermis or muscle restored mating potential (Figure 3A). For *pck-2,* expression in epidermis provided higher rescue than pan muscle rescue, but the difference was not statistically significant. Behavioral rescues by restoring *pck-1* in cholinergic neurons or *pck-2* in all muscles were not surprising, because copulation requires neurons and muscles. However, the rescue from the epidermis-expressed *pck-2* was not expected. The observation suggests that for wild-type *pck-2* to compensate the *pck-1(0)* single mutant deficiency, the epidermis might require PEPCK to provide gluco-/glyceroneogenesis products to neighboring *pck-1*-deficient muscles and neurons.

### Supplementation of Glucose Partially Substitutes the Requirement of PEPCK for Male Copulation Efficiency in Early Adulthood

We next asked how PEPCK from distinct tissues support copulation. Many groups have demonstrated various beneficial functions of PEPCK in well-fed animals. For example, transgenic over-expression of PEPCK provides increased muscle respiratory exchange, mitochondria biomass, behavioral activity, and lifespan extension in mammals and *C. elegans* (Franckhauser et al., 2002, Hakimi et al., 2007, Yuan et al., 2012, Yuan et al., 2016). PEPCK is also essential for mammals and *C. elegans* larvae to survive short and long periods of fasting (Hibshman et al., 2017, Puigserver et al., 2003, Rothman et al., 1991). Enzymatically, PEPCK converts cytoplasmic or mitochondrial OAA into PEP. This reaction achieves two goals: (1) lowering potential mitochondrial ROS production by removing excess substrates from the TCA cycle and (2) diverting excess PEP to the anabolic generation of glucose and glycerol for stored sugar (glycogen and/or trehalose) and triglyceride synthesis (Owen et al., 2002). If the animal experiences fasting, the stored forms of sugar and triglycerides can then be catabolized for further energy usage and building materials. Thus we asked why adult *C. elegans* males require PEPCK for copulation behavior. We hypothesize that they could require PEPCK either for a metabolic protective role or to generate carbon intermediates for sustaining behavioral-related catabolic and anabolic needs.

To address this issue, we supplemented the diets of well-fed *pck-1(0); pck-2(0)* males with D-glucose. If in the context of wild-type behavior, PEPCK diverts excess substrates from the TCA cycle to maintain optimal mitochondrial function, then providing excess carbon into the TCA cycle, via artificial glucose supplementation, should be detrimental for the double mutant. However, if PEPCK is needed for anabolic processes to later distribute catabolic substrates for behavioral execution, then glucose supplementation should compensate for the double mutant's metabolic deficiency.

To evaluate these possibilities, we tested how supplementing their already abundant food availability with D-glucose affects the mutant's copulation ability. We tracked individual males to determine their kinetics for serially impregnating females over a course of 84 h (see Transparent Methods). For this experiment, an individual male was placed with 10 *fog-2* mutant females. Every 12 h, mated egg-gravid females were counted, removed, and replaced with virgin females matched to the age of the male (Figures 3B–3E and S6). The assay allows for the temporal study of male reproductive endurance and behavioral deterioration. During the first 48 h, wild-type and *pck-1(0)* males were able to mate with ∼3–4 partners every 12 h (Figures 3B–3E); however, this was not true for *pck-2* mutant males. Wild type, *pck-1(0)* and *pck-2(0)* males had equivalent mating kinetics during the first 12 h of adulthood, indicating that the two PEPCK paralogs can effectively compensate for each other (Figure 3B). In contrast, the *pck-1(0); pck-2(0)* double mutant displayed a lower ability to serially mate with females. Between 12 and 24 h, *pck-2(0)* mutants began to lag behind wild-type and *pck-1(0)* males, suggesting that by this time, *pck-1*-expressed PEPCK alone can no longer meet the physiological demands of the male. After 24 h, the *pck-2(0)* single and *pck-1(0)*; *pck-2(0)* double mutants were similarly defective for mating.

D-glucose supplementation has been shown to reduce the lifespan of wild-type *C. elegans* hermaphrodites and males (Lee et al., 2009, Schulz et al., 2007, Seo et al., 2018). Our experiments suggest that D-glucose could also have a small negative effect on reproductive-competent wild-type adult males; however, due to the sample size in our assay, the supplemented sugar does not have a statistical effect on their copulation ability (Figure 3B). In contrast, during the first 24 h of adulthood, glucose supplementation improved the copulation performance of *pck-1(0)*; *pck-2(0)* double mutants; they were able to impregnate females at a rate similar to their wild-type counterparts (Figure 3C). After 24 h, sugar supplementation did not sustain the mutants' mating behavior, but the supplementation also did not accelerate behavioral degradation either (Figures 3D and 3E). These observations suggest that early in the wild-type male's adulthood, PEPCK-based gluconeogenesis provides energy to sustain copulation. However, later in the male's adulthood, PEPCK, especially expressed by PCK-2, might be required for other processes such as glyceroneogenesis/triglyceride synthesis to sustain copulation.

### PCK-1 and PCK-2 Expression Levels Are Coordinated and Increase during Aging

**Figure 4:**
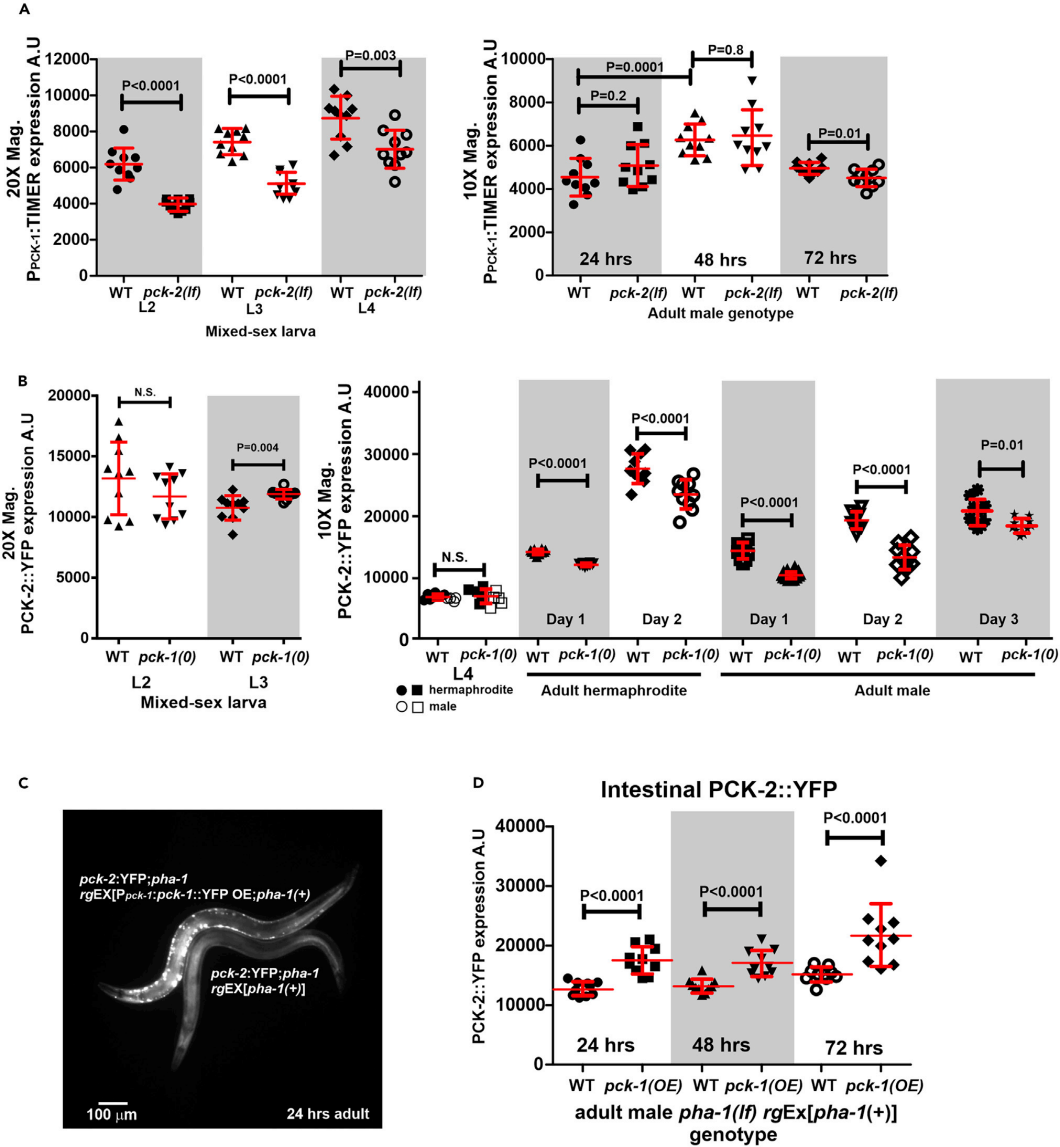
Interactions between PCK-1 and PCK-2 Expression (A) Green emission fluorescence intensity of TIMER expressed from the *pck-1* promoter. (B) Yellow emission fluorescence intensity of whole-body PCK-2::YFP. (C) Fluorescence micrograph of a 24-h *pck-2*::YFP male overexpressing *pck-1*::*YFP* from an extrachromosomal array. The overexpressed (*OE*) PCK-1::YFP forms aggregates in the motor neurons and body wall muscles. A *pck-2*::YFP male is also shown for comparison. Both males contain a *pha-1* mutation and the *pha-1*(*+*)-rescuing plasmid in an extrachromosomal array. (D) Yellow emission fluorescence intensity of intestinal PCK-2::YFP. (A, B, and D) Fluorescence values are in arbitrary units (A.U). For each stage, the number of animals quantified is 10 per strain. Each symbol represents a single male of the specified age. Bars and whiskers represent mean and standard deviation. p values were determined using the Mann-Whitney test. This figure is related to Figure S7.

Given the differential requirement for *pck-1* and *pck-2* in copulation behavior, we asked how PEPCK expression changes between young and mid-aged males. In our previous study, we conducted qRT-PCR analyses that indicated both *pck-1* and *pck-2* transcript levels increased between pooled day 1 and pooled day 2 wild-type males (Guo and García, 2014). Here, we asked if expression from the P_*pck-1*_:TIMER reporter and PCK-2:YFP in individual males agrees with the earlier pooled qRT-PCR results. The fluorescent protein TIMER can be used to estimate the timing of transcription in live cells. The fluorescent molecule initially emits fluorescence in the green wavelength, but after ∼3 h, the molecule stably photoconverts its fluorescence emission to the red wavelength. The changes in green fluorescence intensity can give an approximation of promoter activity (Terskikh et al., 2000). As the green fluorescence emission is transient, the signal should represent actively transcribed transgenes. To quantify the P_*pck-1*_:TIMER expression, we determined the average green fluorescence intensity across the whole ventral cord and ventral body wall muscle region of the male. To quantify total PCK-2::YFP levels, we determined the average yellow fluorescence intensity of the whole male. Consistent with our earlier qRT-PCR findings, P_*pck-1*_:TIMER reporter and PCK-2::YFP expression increases during development and between 24 and 48 h of adulthood. However, by 72 h, the amount of P_*pck-1*_:TIMER expression in males decreases and the accumulation of PCK-2::YFP does not significantly differ from 48-h adult males (Figures 4A and 4B). To isolate the specific tissues that upregulate PCK-2 during adulthood, we set regions of interest over different types of cells and quantified PCK-2::YFP levels. Consistent with its necessity in copulation, the epidermal, but not muscular PCK-2::YFP, level was higher at 48 h, compared with 24 h of adulthood. Interestingly, even though intestinal PCK-2 is not essential for copulation behavior, its expression was also high among different cell types and upregulated at 48 h of adulthood, suggesting additional function of intestinal PCK-2 (Figure S7).

Earlier, we inferred that PCK-2 must compensate for the loss of PCK-1 in the *pck-1(0)* males. PCK-1 is expressed in neurons and body wall muscles; PCK-2 is expressed in the body wall muscles, sex muscles, intestine, and epidermis. As cells likely provide metabolic substrates for each other, we entertained the possibility that changes in one cell might affect another cell's metabolism through metabolite exchange. Thus we asked if differentially reducing PEPCK via mutation in one PEPCK gene will induce a corresponding expression change in its paralog, indicating the interdependency of different cells on their neighbor's metabolic state.

*P*_*pck-1*_:TIMER was integrated close to the *pck-2* locus (∼0.25 map unit), thus we were not able to cross-in the *pck-2*(0) mutation. Instead we used CRISPR/Cas9 to induce a frameshift/premature stop mutation in *pck-2* into P_*pck-1*_:TIMER males. We called this allele *pck-2(lf)* to distinguish it from *pck-2(0).* During larval development, *P*_*pck-1*_:TIMER expression was lower in *pck-2(lf)* compared with wild-type; however, in the first 48 h of adulthood, P_*pck-1*_:TIMER expression for the *pck-2(lf)* males was equivalent to wild-type. After 48 h, P_*pck-1*_:TIMER expression dropped slightly more in the *pck-2(lf),* than in the wild-type (Figure 4A). This result indicates that during wild-type larval development, *pck-1* expression in neurons and muscles is affected by PCK-2 levels in the epidermis, muscles, and intestine, but this dependency is relaxed in adults.

In contrast, when *pck-1(0)* was crossed into *pck-2*::YFP adult hermaphrodites or males, the pattern of expression was reversed. PCK-2::YFP expression levels in *pck-1(0)* L2 to L4 larval animals were similar to wild-type, but during adulthood, PCK-2::YFP levels did not increase to levels of wild-type (Figure 4B). This observation was unexpected. As PCK-2 must compensate for the *pck-1* deletion, we originally predicted *pck-2* expression to be higher in its native tissues or expressed ectopically (such as in neurons). However, the lower expression suggests that in wild-type animals, adult tissues expressing PCK-2 readjust the levels of the enzyme in the direction of PEPCK levels in *pck-1*-expressing tissues; and larval tissues expressing PCK-1 readjust the levels of the enzyme in the direction of PEPCK levels in *pck-2*-expressing tissues.

To test further this idea, we over-expressed *pck-1*::YFP, driven by its own promoter through a transgene; the high expression of PCK-1::YFP led to aggregate formation (Figure 4C). We then asked which direction PCK-2::YFP levels change in the adult intestine, a tissue from which we can isolate fluorescence measurements from PCK-1:YFP. The over-expression of PCK-1 resulted in a coordinated increase in PCK-2::YFP levels (Figure 4D), consistent with the idea that *pck-1* and *pck-2* expression are metabolically coupled and regulated in the same direction.

### Mutation in Subunit A of Succinate Dehydrogenase Leads to Increased PEPCK Expression

**Figure 5:**
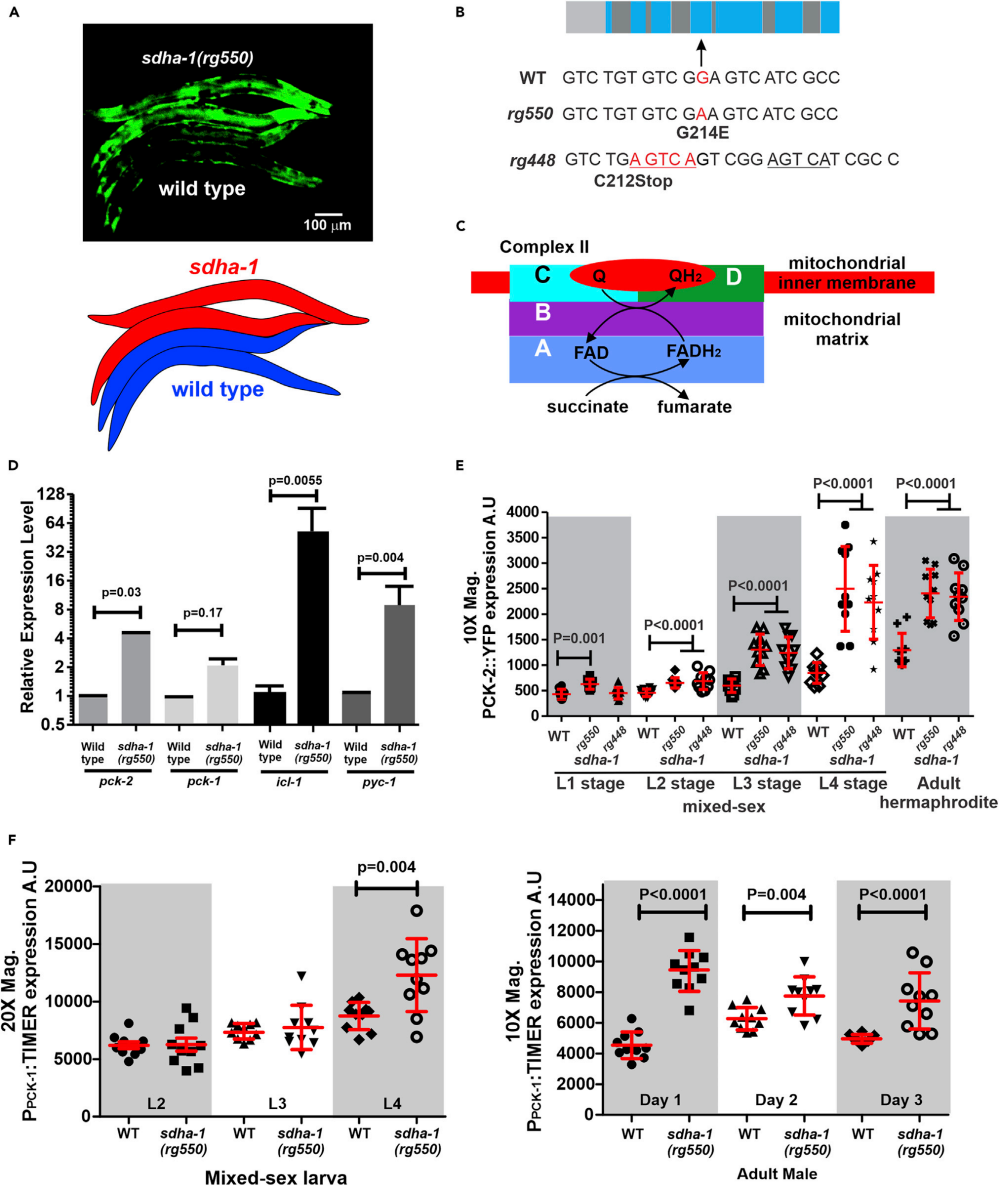
*sdha-1*-Regulating Metabolic Genes Controlling Metabolic Flux of Mitochondria (A) Fluorescence micrograph of PCK-2::YFP expression in 24-h *sdha-1(rg550)* and wild-type hermaphrodites. Below the micrograph is a cartoon showing the worms' positions in the above micrograph. (B) Diagram of the *sdha-1* gene. Light gray is the promoter region, blue regions are the exons, and dark gray regions are the introns. Below the diagram reading left to right 5′ to 3′ are the locations of the *rg550* and *rg448* mutations. The *rg448* mutation is a deletion of a T and a duplication-insertion of the underlined sequence. (C) Cartoon of complex II subunits. (D) qRT-PCR analyses of *pck-2*, *pck-1*, *icl-1,* and *pyc-1* cDNA levels in 12- to 24-h wild-type and *sdha-1(rg550)* males. Columns and whiskers show mean and standard error of the mean of three biological replicates; each biological replicate consists of pooled 500 males. p values were determined using the paired t test between wild-type and mutant RNA samples that were processed the same day. (E) Yellow emission fluorescence intensity of whole worm PCK-2::YFP during multiple developmental stages of wild-type and *sdha-1* mutants. (F) Green emission fluorescence intensity of TIMER expressed from the *pck-1* promoter during multiple developmental stages of wild-type and *sdha-1(rg550)* mutants. (E and F) Fluorescence values are in arbitrary units (A.U). For each stage, the number of animals quantified is 10 per strain. Each symbol represents a single male of the specified age. Bars and whiskers represent mean and standard deviation. p values were determined using the Mann-Whitney test. This figure is related to Figures S8 and S9.

We hypothesized that *pck-1* and *pck-2* expression increases as a response to a change in the male's physiology; however, we did not know what metabolic alterations would induce PEPCK levels to increase. To address this issue, we performed an ethyl methanesulfonate (EMS) mutagenesis on *pck-2*::YFP animals to identify what genetic changes could increase PCK-2::YFP expression. Our screen identified the *rg550* missense allele in the gene *sdha-1* (Figure 5A). The mutation creates a glycine to glutamic acid change in the fourth exon (Figure 5B). The *C. elegans sdha-1* and its paralog *sdha-2* encode the A subunit of the multisubunit mitochondrial respiratory Complex II. Under aerobic conditions, this complex functions in both the TCA cycle and the electron transport chain to oxidize succinate into fumarate and participate in ubiquinone reduction (Figure 5C). Under anaerobic conditions, subunits in this complex can also participate in the reduction of fumarate back to succinate (Kuang and Ebert, 2012, Kuramochi et al., 1994, McElwee et al., 2006, Takamiya et al., 1999). Consistent with the increase in PCK-2::YFP accumulation, qRT-PCR analysis indicated that the *sdha-1* mutant also express more *pck-2* transcripts (Figure 5D). *pck-1* transcript level was also slightly higher in the mutant, but not statistically different, likely due to the low number of independent biological trials (n = 3) (Figure 5D).

Animals that are homozygous for the *sdha-1(rg550)* allele displayed multiple abnormal phenotypes (Figure S8), similar to reported RNA interference (RNAi) phenotypes and mutations in other electron transport chain molecules (Huang and Lemire, 2009, Ishii et al., 1990, Mathew et al., 2016). We recorded the following defects in the *sdha-1* mutants: their mitochondria were smaller and less networked than wild-type animals (Figures S8A and S8B), they consumed oxygen at a slower rate (Figure S8C), their larval developmental rate between L2 to L3 stage was slower (Figure S8D), they moved slower (Figure S8E), developing L4 males failed to remodel their anal depressor muscle and are not capable of copulation (Figures S8F and S8G), they were partially resistant to the reactive oxygen-producing poison paraquat (Figure S8H), and adult hermaphrodites retained eggs in their uterus (Figure S8I). These phenotypes indicate that mitochondrial function is compromised, detrimentally impacting development and behavior.

Hermaphrodites that are heterozygous for *sdha-1* grossly appeared wild-type, suggesting that *rg550* might be a recessive loss-of-function allele; however, as the *sdha-1(rg550)* allele is a missense mutation, the mutant phenotypes might be due to neomorphic functions caused by the amino acid change. We addressed this possibility using CRISPR/Cas9 to induce into wild-type *sdha-1* an insertion/deletion mutation (*rg448*) in the general position of *rg550*. The *rg448* lesion creates a premature stop, followed by a frameshift change lesion (Figure 5B). The developmental and behavioral defects induced by *rg448* resembled animals containing the *rg550* allele. Similarly, with respect to whole worm PCK-2::YFP increase, the *rg448* and *rg550* lesions promoted *pck-2* expression through every stage of development (Figure 5E). Thus *rg550*-induced point mutation likely disrupts SDHA-1 function.

We also examined P*pck-1*:TIMER expression in *sdha-1(rg550)* mutants, as the qRT-PCR experiment measuring *pck-1* expression was slightly higher, albeit not statistically different between wild-type and mutant (Figure 5D). Similar to PCK-2::YFP, we found that P*pck-1*:TIMER expression increased in adulthood (Figure 5F). These observations are consistent with the idea that the animal modulates PCK-1 and PCK-2 levels in the same direction. We also attempted to use CRISPR/Cas9 to generate a mutation in *sdha-2*, the paralog of *sdha-1*; however, we were unsuccessful at obtaining viable mutants. This might be because unlike *sdha-1*, *sdha-2* is essential for the survival of the worms.

**Figure 6:**
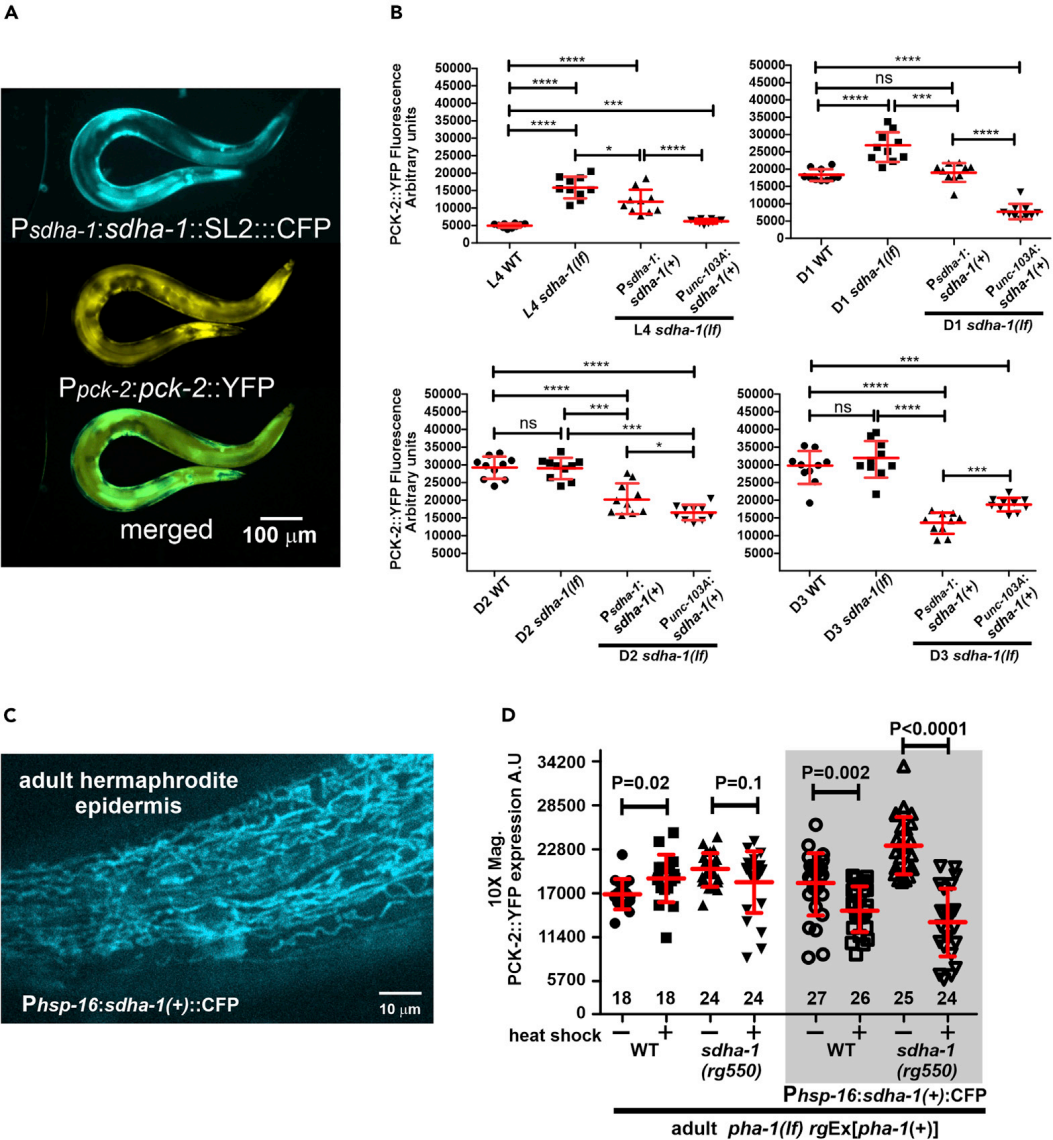
SDHA-1 Inhibition on PCK-2 Expression in Hermaphrodites and Males (A) Fluorescence micrographs showing a PCK-2::YFP male expressing *sdha-1* and SL2 *trans*-spliced CFP from the *sdha-1* promoter. (B) Yellow emission fluorescence intensity of whole-animal PCK-2::YFP during different adult ages. Fluorescence values are in arbitrary units (A.U). Symbols represent individual wild-type, *sdha-1(rg550),* and transgenic *sdha-1(rg550)* transgenic males that express *sdha-1(+)* from its native promotor (*sdha-1* promoter) or a muscle promoter (*unc-103A* promoter). For each stage, the number of animals quantified is 10 per strain. Each symbol represents a single male of the specified age. Bars and whiskers represent mean and standard deviation. p values were determined using the Mann-Whitney test. (C) Fluorescence micrograph showing epidermal CFP-expressing mitochondria from a heat-shocked hermaphrodite that expresses CFP fused to SDHA-1. (D) Yellow emission fluorescence intensity of whole animal PCK-2::YFP in 18- to 24-h adult males. Males were heat shocked at L4 stage to either induce transgenic *sdha-1*:CFP expression or test if heat shock artificially changed PCK-2::YFP levels. Fluorescence values are in arbitrary units (A.U). Symbols represent individual wild-type *sdha-1(rg550)* and transgenic wild-type and *sdha-1(rg550)* males that express *sdha-1::CFP* from a heat shock promoter (*hsp-16* promoter). Numbers of animals quantified are listed at the bottom of the graph. Bars and whiskers represent mean and standard deviation. p values were determined using the Mann-Whitney test.

To verify that *sdha-1* mutant phenotypes were not caused by an unknown mutation in our strains, we tested if the *sdha-1*(*rg550*) missense mutation can be rescued by an extrachromosomal array expressing *sdha-1(+)* and a separate SL2 splicing CFP from the *sdha-1* promoter. A 500-bp region upstream of the first *sdha-1* ATG start codon drives *sdha-1* expression in the pharynx, epidermis, body wall muscles, and intestine; these are tissues that also express the *pck-2* gene product (Figure 6A). From casual observation, we found that expression from the extrachromosomal array complements the *sdha-1* growth and behavioral defects in both sexes. Focusing on adult males, we also found that the extrachromosomal array reduces the *sdha-1*(*rg550*)-induced PCK-2 increase from L4 stage to the third day of male adulthood; in some mutant males, *pck-2* expression was even lower than in wild-type (Figure 6B). These observations confirm that the *rg550* missense mutation reduces the function of SDHA-1 and promotes PCK-2 expression.

We noticed that males that were mosaic for the transgene (as determined by patchy CFP expression throughout the worm) had uniformly lowered PCK-2::YFP expression in the mosaic tissue; this observation prompted us to hypothesize that tissues containing a functional mitochondrial Complex II system might partially modulate the metabolism of neighboring tissues. To test this possibility, we generated an extrachromosomal array that selectively expresses *sdha-1(+)* in the body wall and sex muscles (from the *unc-103A* promoter) of *sdha-1(rg550)* mutants. We found that similar to expression from its own promoter*, sdha-1(+)* expression from the body wall muscle also had a cell non-autonomous effect on PCK-2::YFP levels in the epidermis, intestine, and pharyngeal muscles through several days of adulthood (Figure 6B). This result is consistent with our earlier experiment (Figure 4D) showing that altering PEPCK level in one tissue changes PEPCK levels in neighboring ones.

In the transgenic *sdha-1(+)-*containing mutants, *sdha-1(+)* was chronically overexpressed throughout development. However, this experiment did not differentiate if the reduction in *pck-2* expression was indirectly due to restoration of mitochondrial function at a specific stage in development, or due to an immediate acute response to expressed SDHA-1 levels. Thus we asked if acute expression of *sdha-1(+)* from a heat shock promoter at L4 stage can immediately alter PCK-2::YFP expression in early adult males. Instead of using the *trans*-splicing *sdha-1(+)*:SL2::CFP plasmids (Figures 6A and 6B), we constructed *sdha-1(+)* directly fused to CFP at the C terminus, to visualize whether the expressed protein went to the mitochondria (Figure 6C). We found that heat shock expression of *sdha-1(+)*:CFP in L4 animals reduces PCK-2::YFP expression in 18- to 24-h adult wild-type and *sdha-1(rg550)* (Figure 6D). Thus, acute alteration of SDHA-1 levels results in the opposite change in PCK-2 levels; however, neither the heat shock nor the endogenous 500-bp *sdha-1* promoter expressed *sdha-1(+)*:CFP rescue *sdha-1* behavioral and developmental defects (casual observation). As the *sdha-1(+)*:CFP fusion from those constructs are stable and transgenically overexpressed, we reasoned that the failure of rescue is not likely from lack of production, instead the CFP, tagged to SDHA-1, likely interferes with a function of the subunit, which is independent of its role in modulating PEPCK levels.

### PEPCK Regulates Fuel Usage and Energy Metabolism in *sdha-1* Mutant and Wild-Type

As *sdha-1* mutants display defective mitochondria and upregulate PEPCK, we hypothesized that genes, which divert substrates out of the oxidizing steps of TCA cycle and promote OAA production, should also be highly expressed in the mutant. *pyc-1*-encoded pyruvate carboxylase converts pyruvate to oxoaloacetate, and the bifunctional *icl-1*/*gei-7*-encoded isocitrate lyase/malate synthase enzyme promotes succinate and malate production directly from isocitrate; both these enzymes are expressed in the mitochondria (Liao and Freedman, 2001, Liu et al., 1995). Consistent with our hypothesis, qRT-PCR data indicated that *sdha-1* mutants hyper-express *pyc-1* and *icl-1* (Figure 5D). However, although the anabolic genes *pck-1*, *pck-2*, *icl-1,* and *pyc-1* are upregulated, *sdha-1(rg550)* mutants accumulated less glycogen than wild-type and the *pck-2(0)* mutant (Figures S9A and S9B). Moreover, the *sdha-1(rg550)* mutants also accumulated less intestinal neutral lipid droplets than wild-type, but more than the *pck-2(0)* mutant. Interestingly, under starvation conditions, the *sdha-1(rg550)* mutant retained more lipids than wild-type, and this property depended on functional *pck-2* (Figure S9C). We hypothesize that instead of stockpiling lipids and carbohydrates, the *sdha-1(rg550)* mutants use PCK-1, PCK-2, ICL-1, and PYC-1 to generate the necessary fuel for immediate catabolism or to ration fuel under food-limiting conditions.

### ATP Production in Wild-Type, *sdha-1,* and PEPCK Mutants

**Figure 7:**
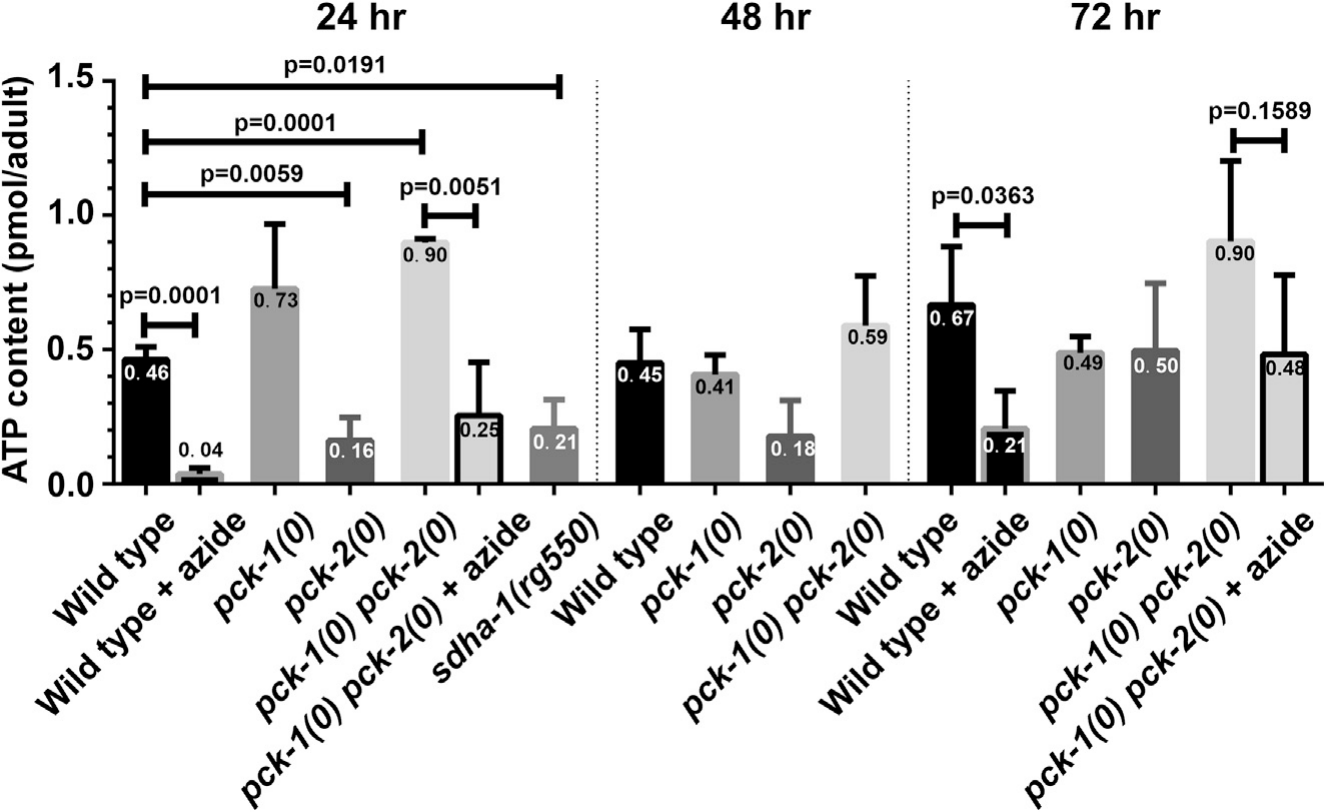
ATP Levels in Wild-Type, *sdha-1,* and PEPCK Mutants ATP content of wild-type, PEPCK, and *sdha-1* mutant males determined using whole-worm luciferase assay at days 1, 2, and 3 of adulthood. Columns and whiskers show mean and standard deviation of the mean of three biological replicates; each biological replicate consists of pooled 20 males. Numbers with the columns represent the average ATP content (pmole/adult). p values were determined using the t test.

**Figure 8:**
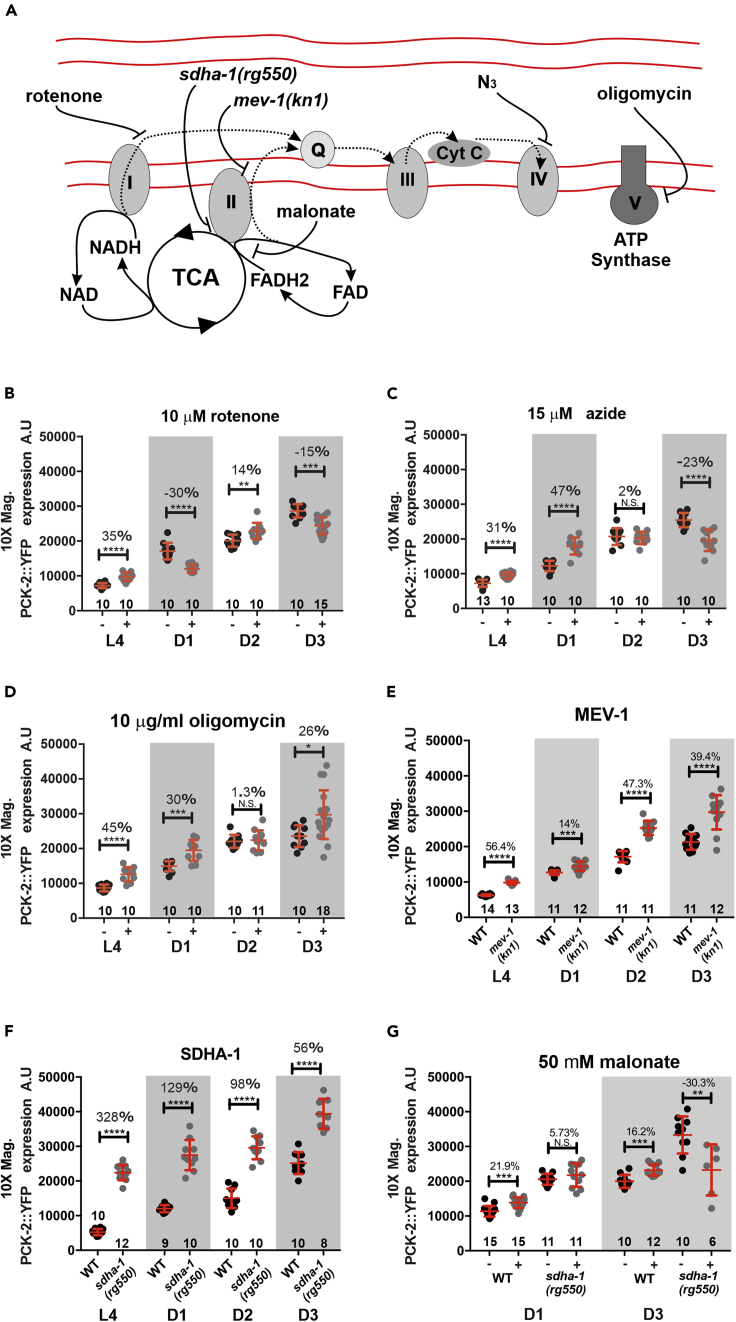
Influences on PCK-2 Expression by Perturbing Mitochondrial Functions (A) Cartoon of the mitochondrial electron transport system, and site of action for mutations and electron transport chain toxins. (B–G) (B) Yellow emission fluorescence intensity of PCK-2::YFP in L4 and day 1 (D1) through day 3 (D3) hermaphrodites grown in the absence (−) or presence (+) of rotenone (B), azide (C), oligomycin (D), and malonate (G) or containing mutations in *mev-1(kn1)* (E) and *sdha-1(rg550)* (F and G). Yellow emission fluorescence intensity of day 1 (D1) and day 3 (D3) wild-type or *sdha-1(rg550)* PCK-2::YFP hermaphrodites grown in the absence (−) or presence (+) of malonate. Bars and whiskers represent mean and standard deviation. (B–G) Numbers above the bars represent percent increase or decrease (−) between treated or mutant animals relative to the untreated or wild-type reference. p values were determined using the Mann-Whitney test; ∗p < 0.05, ∗∗p < 0.01, ∗∗∗p < 0.001, ∗∗∗∗p < 0.0001. Numbers of animals quantified are listed at the bottom of the graphs.

To further dissect the energy metabolism of aging animals, we determined the whole worm ATP content using a luciferase assay (Figure 7). Wild-type and *pck-1(0)* males maintained relatively constant ATP throughout the first 3 days of adulthood. As expected, disrupting mitochondrial function on day 1 via *sdha-1* mutation or by acutely exposing day 1 and day 3 wild-type or day 1 *pck-1(0); pck-2(0)* double mutant males to the mitochondrial poison sodium azide (NaN_3_) significantly lowered the ATP content; the remaining ATP was likely generated by substrate-level phosphorylation, such as glycolysis. NaN_3_ inhibits ATP generation by blocking electron transfer between cytochrome *c* and complex IV (Figure 8A) (Palmieri and Klingenberg, 1967, Wilson and Chance, 1966). In contrast to wild-type and *pck-1(0)* males, day 1 *pck-2(0)* single mutants showed significantly lower ATP content than wild-type, indicating that unregulated PCK-1 function, in the absence of *pck-2*, abnormally lowers ATP levels (Figure 7).

Surprisingly, the *pck-1(0)*; *pck-2(0)* double mutant had higher ATP content than wild-type, *pck-1(0)* and *pck-2(0)* single mutants (Figure 7). In addition, treating the double mutant with NaN_3_ variably reduced the amount of ATP comparable to wild-type (wild-type, 90% ± 10% reduction versus double mutant, 72% ± 22% reduction) suggesting that either or both mitochondrial ATP production and substrate level phosphorylation are abnormally higher in the mutant. The contribution of substrate level phosphorylation on the double mutant's ATP production is most evident on day 3, because NaN_3_ did not significantly reduce the ATP levels. Possibly, the prolonged lack of accumulated gluco-/glyceroneogenesis products (i.e., fats) causes day 3 *pck-1(0)*; *pck-2(0)* double mutant energy production to shift away from mitochondrial respiration.

If a function of PEPCK is to divert metabolic intermediates out of the mitochondria, then early-on, lacking both PEPCK paralogs could initially result in aberrant mitochondria with more TCA cycle intermediates, and thus more ATP production. In addition, compensation for the reduction in PEPCK could also result in increased substrate-level phosphorylation through altered catabolism of ingested or scavenged five-carbon sugars. However, these mechanisms to increase ATP production are not sufficient to offset defective gluco-/glyceroneogenesis, because dietary glucose supplementation is required to partially alleviate the double mutant's copulation defect. Likely, the temporal and spatial production of energy cannot be fully restored by compensatory mechanisms.

### Electron Transport Chain Poisons Increase PEPCK Expression, but Less Than *sdha-1* Deficiency

In the *sdha-1* male and hermaphrodite mutants, the increase in *pck-1* and *pck-2* expression could be due to a reduction of a specific function of succinate dehydrogenase, due to non-specific mitochondrial dysfunction, or due to a general response to mitochondrial stress, such as unfolded protein accumulation or amino acid limitation (Méndez-Lucas et al., 2014). We addressed these possibilities by asking if impairing other mitochondrial electron transport chain components can also increase PEPCK levels to the same level as the *sdha-1* mutations. As the *sdha-1* mutations affect PEPCK expression in both sexes, we simplified our quantification by just analyzing hermaphrodites. Hatched L1 hermaphrodite worms were grown until adulthood in the presence of the mitochondrial poison rotenone (blocks electron transfer between complex I and ubiquinone), NaN_3_, or oligomycin (blocks complex V ATP synthase) (Figure 8A) at concentrations that slowed development and caused 5% to 10% lethality. Exposure to rotenone (Figure 8B), NaN_3_ (Figure 8C), and oligomycin (Figure 8D) had modest variable effects on *pck-2* expression over the first 3 days of adulthood.

Unlike the mitochondrial poisons used to disrupt the electron transport chain, the *sdha-1* mutation also affects the TCA cycle by reducing the formation of fumarate from succinate. To specifically target the electron transport function of complex II, we tested if a loss-of-function point mutation in subunit C of complex II, *mev-1(kn1)*, can also increase PCK-2::YFP expression. Subunit C participates with subunit D of complex II to reduce ubiquinone. Worms containing the *mev-1(kn1)* point mutation have reduced mitochondrial respiration and are hypersensitive to the electron-accepting ROS generator paraquat. These defects indicate that the *mev-1 (kn1)* mutation disrupts electron flow from complex II and leaks electrons away from the electron transport chain (Ishii et al., 1990) (Ishii et al., 1998, Yanase et al., 2002); however, similar to the electron transport chain poisons, the *mev-1(kn1)* mutation (Figure 8E) does not increase *pck-2* levels as high as the *sdha-1(rg550)* allele (Figure 8F). This observation suggests that reduced succinate dehydrogenase's catalytic activity in the TCA cycle might also contribute to increased PEPCK expression.

We use the competitor malonate to address whether interfering with the TCA cycle can also increase PCK-2::YFP levels. Malonate should reversibly compete with succinate for binding to the SDHA-1 and SDHA-2 active site on complex II; thus the competitor should interfere with the oxidation of succinate into fumarate (Krebs and Eggleston, 1940). When L3 worms were grown on UV-killed OP50, supplemented with 50 mM malonate, they expressed slightly more PCK-2::YFP than those fed with UV-killed OP50 (Figure 8G), but not as much as the *sdha-1(rg550)* mutants (Figure 8F). The moderate malonate-induced increase in PCK-2::YFP expression could be due to insufficient competition between the exogenously added malonate with endogenous succinate levels. Nonetheless, taken with the results from the electron transport chain inhibitors, reducing succinate dehydrogenase's activities to both the electron transport chain and TCA cycle contributes to increased PCK-2 levels.

As we showed that malonate can artificially increase PCK-2 in wild-type animals, we asked if the competitor can further increase PCK-2 levels in the *sdha-1* mutant, by interfering with the SDHA-2 paralog. We casually observed that when L3 *sdha-1(rg550)* mutants were grown on UV-killed OP50 supplemented with malonate, they developed 4–5 h slower than their control cohorts and many died by day 3 as either developmentally stalled L4 animals or as adults with internal hatched worms. Although malonate had a detrimental effect on succinate dehydrogenase activity in the *sdha-1* mutant, PCK-2::YFP did not further increase on day 1 or on day 3; indeed, less PCK-2::YFP was measured on day 3, likely due to malonate toxicity on viability (Figure 8G). These observations indicate that the worm is limited by how much PEPCK can be expressed after insult to succinate dehydrogenase function.

## Discussion

In this work, we explore how N2 male *C. elegans* copulation fitness is metabolically sustained during the first few days of adulthood. The copulation fitness of N2 males, raised at 20°C under abundant *E. coli* food conditions, drops after 48 h of adulthood; by 96 h, most males are impotent (Guo et al., 2012, Hodgkin and Doniach, 1997). N2 males in laboratories are mainly maintained for use in occasional out-crossings. The amount of selection pressure for maintaining male-propagating lines likely differs from laboratory to laboratory, depending on idiosyncratic research demands. The abbreviated copulation window for males can be a consequence of decades-long relaxed laboratory conditions that researchers use to propagate the N2 strain (Hodgkin and Doniach, 1997). In the laboratory, worms are restrained to *E. coli* lawns 3–4 cm in diameter. Under standard conditions, worms can develop from fertilize egg to reproducing adult in ∼65 h (Byerly et al., 1976). Owing to food overabundance and a constrained habitat, pressure to maintain copulatory fitness after 48 h of adulthood is not strong. For example, if a male copulates soon after it reaches adulthood, and it continues to copulate every 2–3 h afterward, before the third day of adulthood, it will be overwhelmed from the multitudes of its own male progeny competing for mates. We do not rule out that altruistic copulatory senescence could be occurring in the adult to promote reproductive fitness for its progeny, as has been computationally modeled for aging senescence and population fitness (Lohr et al., 2019); however, as the male can sire many progeny males after each ejaculation (Murray et al., 2011), we favor the more pedestrian idea that laboratory conditions pressure the wiring and fueling of the N2 male neuromuscular circuitry to promote copulatory competition between age-matched brothers, rather than between father and sons. In the wild, temperature and food conditions for nematodes are more variable, and the range for dispersal is not so constrained, which might select for males of natural strains to have longer stamina and duration for siring progeny. Accordingly, males of wild *C. elegans* strains have been demonstrated to mate longer into adulthood (Hodgkin and Doniach, 1997, Wegewitz et al., 2008).

Although well-fed N2 males' sexual potency drops between 48 and 72 h, we found that before reproductive decline, 24- and 48-h adults are similar in their competitive fitness. Our earlier studies found that expression of certain male metabolic genes changes within this period, raising the possibility that the well-fed male adjusts its metabolism to maintain their reproductive fitness. Between 24 and 48 h of adulthood, genes that direct the glyoxylate cycle, gluco-/glyceroneogenesis, lipid synthesis, as well as glycolysis and fatty acid oxidation increase in expression. This gene expression profile suggests that the 24- to 48-h adult males are shifting intermediates from the mitochondrial TCA cycle to anabolic processes (Guo and García, 2014). One type of enzyme that increases between 24 and 48 h of adulthood is *pck-1-* and *pck-2*-encoded PEPCK, an enzyme that promotes metabolic remodeling (DeBerardinis et al., 2007, Montal et al., 2015) and catalyzes a rate-controlling step of gluco-/glyceroneogenesis (Rognstad, 1979).

We found that of the two functional *C. elegans*' PEPCKs, PCK-1 is expressed in neurons and in body wall muscles, whereas PCK-2 is expressed in body wall muscles, sex muscles, enteric muscles, intestine, epidermis, and pharyngeal muscles. With respect to mating behavior, PCK-1 function is required in the neurons, whereas PCK-2 function is required in the epidermis. In higher animals, there is a cytoplasmic and mitochondrial form of PEPCK (Ballard and Hanson, 1967, Nordlie and Lardy, 1963). The cytoplasmic form uses GTP to convert OAA, derived from pyruvate carboxylase or the malate-aspartate shuttle, into PEP, GDP, and carbon dioxide. The mitochondrial form does a similar catalysis, but can participate in altering the flow of TCA cycle intermediates to generate mitochondrial PEP under conditions of glucose limitation (Vincent et al., 2015). In contrast to higher animals, we find both PCK-1 and PCK-2 to be cytoplasmic in *C. elegans.*

Previously, PCK-1 has been shown to promote fitness, lifespan, longevity, and starvation survival by enhancing carbohydrate biosynthesis, glucose consumption, oxidative metabolism, autophagy, and retarding mitochondrial aging (Hibshman et al., 2017, Yuan et al., 2016). Here, we find that PCK-2 has a more essential role than PCK-1 for sustaining male copulation fitness. Males that are mutant for *pck-1* do not have an observable fitness deficit, whereas *pck-2* mutants copulate less competitively and prematurely decline in the ability to sire progeny. However, PCK-1 is still involved in copulation, because early in adulthood, *pck-1*; *pck-2* double mutant males are less behaviorally fit than *pck-2* mutants. The synthetic double mutant phenotype indicates that PCK-2 function can fully compensate for the *pck-1* deficiency, whereas PCK-1 can partially compensate for *pck-2* deficiency. The *pck-1; pck-2* double mutant males have a high rate of spontaneous spastic sex muscle contractions, which is aggravated by repeated copulation attempts. Based on previous research, this defect is usually associated with neural muscular hyperexcitability in mutants of *ether-a-go-go* K^+^ channels, such as *unc-103* and *egl-2* (Garcia and Sternberg, 2003, LeBoeuf et al., 2007). In this study, we found that the *pck-1; pck-2* double mutant's phenotype is correlated with a decrease in *ether-a-go-go* EGL-2 K+ channel expression. Previous research demonstrated that transient starvation and aging can upregulate EGL-2 activity through an UNC-42/CAMKII- and DAF-16/FOXO-independent DAF-2/insulin receptor-like pathway (LeBoeuf et al., 2007, LeBoeuf et al., 2011, Reiner et al., 2006). The correlation between EGL-2 and PEPCK expression suggest that similar mechanisms might be adopted here as *ether-a-go-go* K+ channels regulate membrane thresholds as an adaptive response to age-related changes in the metabolism.

We find that PEPCK's function is essential for ATP homeostasis, because *pck-*2 or *pck-1; pck-*2 mutations alter ATP contents and their response toward NaN_3_. The higher-than-normal ATP level in the *pck-1(0); pck-2(0)* double mutant suggests that the mutant does not suffer from overall ATP depletion, rather they are defective in utilizing metabolites or energy production at specific sites. We hypothesize that PEPCK facilitates redistribution among different tissues to accommodate such needs. This hypothesis is consistent with the benefit of glucose supplementation on increasing the double mutants' copulation ability. Therefore, the possible requirement for PEPCK in day 1 and day 2 wild-type copulation behavior is to provide gluco-/glyceroneogenic substrates that ultimately sustain mitochondrial energy production for competitive fitness. PEPCKS′ supportive role in maintaining mitochondrial function is also observed on day 3, because the *pck-1(0); pck-2(0)* mutant alters its reliance of ATP production from mitochondria.

The *C. elegans* epidermis is composed of several multinucleated tissues that are in contact with muscles and neurons. The epidermis secretes the worm's external collagen-based cuticle that acts as the worm's elastic exoskeleton and protects its internal cells from the outside environment. However, others have implicated that the epidermis is important in providing carbohydrates, amino acids, fatty acids, and growth signals to neighboring cells for promoting development through the different larval stages, for promoting recovery from starvation, and for extending lifespan (Dalton and Curran, 2018, Ewald et al., 2016, Fukuyama et al., 2015, Kaletsky et al., 2018, Kennedy et al., 2013, Saudenova and Wicky, 2018, Son et al., 2018). Our results suggest that at least between 24 and 48 h of adulthood, the epidermis plays a critical role in supplementing fuel to the neural musculature for the male to sustain reproductive behavior.

The intromission circuitry is composed of multiple interconnected sensory-motor neurons that induce fast and slow twitch-like responses in the copulatory spicule muscles. Upon contact with the vulva, the post-cloacal sensory-motor neurons promote continuous high-frequency muscle contractions that produce repetitive spicule thrusts at the vulva. When the spicules partially penetrate the vulval slit, the SPC proprioceptive motor neurons induce sustained spicule muscle contraction, which forces the spicules through the vulva. The SPD and SPC sensory neurons, in conjunction with the SPC motor neurons, then regulate ejaculation (LeBoeuf et al., 2014). Despite the expression of *pck-1* in neurons and *pck-2* in muscles, we hypothesize that the male epidermis must provide additional metabolic investment for maintaining membrane thresholds used in triggering rapid on-and-off motor responses during extended stretches of copulation. Our data indicate that in *pck-1* mutant males, epidermal PCK-2-mediated gluco-/glyceroneogenesis can provide fuel (either as glucose or the 1,1-glycosidic linkage disaccharide trehalose) to neurons and muscles for mating behavior. In contrast, cell-autonomous PCK-1-mediated processes in neurons or PCK-2-supplied PEPCK in the sex muscles cannot provide sufficient energy for maintaining behavioral fitness after 12–24 h of adulthood.

The rise in *pck-1* and *pck-2* expression, along with genes involved with glyoxylate, glycolytic, fatty acid oxidation, and fatty acid biosynthesis pathways, indicates a change in the 24- to 48-h well-fed adult male physiology (Guo and García, 2014). The decay in male copulation fitness can be delayed by raising males in antioxidants, suggesting that very early adult processes might already be stressed and metabolic alterations might be compensating for the decline (Guo and García, 2014). The EMS mutagenesis screen identified the mitochondrial succinate dehydrogenase subunit A as a potential molecule whose activity might be purposely down-regulated or subtly be in decline in the early adult. Succinate dehydrogenase participates in both the TCA cycle and electron transport chain. Subunit A is covalently bound to FAD and couples the oxidation of succinate and the reduction of FAD. Electrons then move through Fe-S clusters in the B subunit, where they then reach the ubiquinone-binding site at the subunit C and D interface (Moosavi et al., 2019). Our observations that loss-of-function mutations in *sdha-1* increase and *sdha-1* over-expression decreases *pck-2* expression suggest that for well-fed animals, the levels of anabolic processes might be inversely related to mitochondrial function. Others have observed that RNAi-mediated reduction of complex I NADH/ubiquinone oxidoreductase subunit V1 and complex II succinate dehydrogenase subunit B and D promoted *pck-1* RNA expression (Zuryn et al., 2010). Similarly, we also noticed modest changes to *pck-2* expression after incubation with mitochondrial electron transport chain energy poisons. However, as these effects are not as pronounced compared with the *sdha-1* deficiency, we speculate that for wild-type animals, succinate dehydrogenase's dual participation in the TCA cycle and electron transport chain play a more pivotal role in modulating anabolic and catabolic processes.

Animals containing the loss-of-function *sdha-1* alleles are viable, but do have cell non-autonomous developmental and behavioral deficiencies. Viability of the *sdha-1* mutant indicates that its *sdha-2* paralog can provide function to the TCA cycle, albeit at reduced capacity. Similar to mutants that are defective in the mitochondrial complex I and the ATP synthase, *sdha-1* mutants developmentally stall at the L2/L3 stage (Rea et al., 2007, Tsang and Lemire, 2002, Tsang et al., 2001). Like other larval developmental genes (Fay and Han, 2000), developmental arrest could be due to depletion of maternal *sdha-2.* However, developmental arrest is temporary, and we speculate that the *sdha-1* mutants can eventually express enough *sdha-2* to progress the animals into fertile adulthood.

Our acute *sdha-1* rescue experiments suggest that increased PEPCK expression is not due to enzyme accumulating during developmental stalling; however, we cannot rule out that the mechanism of increasing PEPCK expression via *sdha-1* mutation is distinct from the increased PEPCK expression observed in aging wild-type males. Because of similarities between 48-h wild-type and *sdha-1* mutant males, we favor the possibility that alteration in mitochondrial function is occurring in the former. Our previous study found that mRNAs that encode for multiple mitochondrial metabolic proteins, including the *mev-1*-encoded succinate dehydrogenase subunit C, are significantly less in 48-h-old wild-type males (supplementary file 1 in (Guo and García, 2014)). In contrast, the *pyc-1-encoded* pyruvate carboxylase (Liao and Freedman, 2001) and the bifunctional *icl-1* encoded isocitrate lyase/malate synthase enzyme (Liu et al., 1995) are upregulated in 48-h wild-type males (Guo and García, 2014). Similarly, both genes are also increased in *sdha-1* males. Pyruvate carboxylase catalyzes pyruvate into OAA, and isocitrate lyase participates in the glyoxylate pathway, which bypasses TCA cycle decarboxylation steps and promotes malate to OAA production. The coordinated increase of *pck-1*, *pck-2*, *pyc-1,* and *icl-1* in wild-type and *sdha-1* mutants suggests that anabolic processes are enhanced when mitochondrial processing of intermediates into the TCA cycle is altered.

Most studies concerning gluco-/glyceroneogenesis and the glyoxylate pathway in *C. elegans* have been in the context of environmental stress handling and postreproductive aging. The disaccharide trehalose is one of many possible end products of gluco-/glyceroneogenesis. Trehalose, internally synthesized or externally fed, has been shown to be a stress protectant against neurodegeneration, osmotic stress, temperature fluctuations, and dehydration, as well as being a mobile metabolizable sugar (Lee et al., 2018, Seo et al., 2018). The glyoxylate pathway genes and gluco-/glyceroneogenesis genes are highly expressed during early embryogenesis, larval starvation, dauer development, and dietary restriction (Castelein et al., 2008, Hibshman et al., 2017, Holt and Riddle, 2003, Liu et al., 1997). Common between these conditions is that the animals are deprived of external food and thus are metabolizing internal stores. In previous studies, we also showed that transient starvation between late L4 stage and 12 h of adulthood can reduce mutation-induced and aging-related neural muscular hyperexcitability (Gruninger et al., 2008, Guo et al., 2012, LeBoeuf et al., 2007). Transient starvation can also extend the sexual potency of a wild-type aging male. However, the mating extension comes at a fitness cost when the male is younger; a young well-fed male will successfully transfer sperm into a mate before an age-matched starved/refed male during a copulation competition (LeBoeuf et al., 2011). In this report, the 24- to 48-h males are not food-stressed, but the changes in the levels of their gluco-/glyceroneogenesis genes suggest that they are adapting some elements of a food-stressed metabolism to sustain competitive behavioral fitness. The activity profile of feeding behavior with respect to mating behavior is poorly studied in the *C. elegans* male, thus future studies can explore if the male modulates its feeding behavior to promote a caloric restriction-like metabolism. In addition, although our data suggest that these males require gluco-/glyceroneogenesis to maintain fuel for competitive copulation, we do not rule the possibility that trehalose production might provide catabolic-independent stress protective functions to sustain mating behavior.

### Limitations of the Study

In this study, we report that 24- to 48-h adult male *C. elegans* requires the continual expression of neural and epidermal PEPCK to sustain competitive copulation behavior. Many of our interpretations hinge on the assumption that in the knock-in animals, fluorescence intensity of YFP-tagged PEPCK reflects similar PEPCK levels in non-tagged wild-type animals. We acknowledge that the YFP tag might interfere with PEPCK turnover and the increased fluorescent signal during aging might be due to accumulation of non-functional protein. Nonetheless, the continual accumulation of the fluorescently tagged YFP indicates that the older males are still synthesizing the enzyme. We do not interpret the increase in fluorescent signal as the males are participating in more gluco-/glyceroneogenesis than in any other life stage of the male.

## Methods

All methods can be found in the accompanying Transparent Methods supplemental file.

## Supplemental Information

Document S1. Transparent Methods and Figures S1–S9
