# Supplementary material for: Succinate Dehydrogenase-Regulated Phosphoenolpyruvate Carboxykinase Sustains Copulation Fitness in Aging C. elegans Males
Source: iScience. 2020 Mar 19;23(4):100990. doi: 10.1016/j.isci.2020.100990 (PMC7115159; doi:10.1016/j.isci.2020.100990)
Supplement: Document S1. Transparent Methods and Figures S1–S9 [file mmc1.pdf]

iScience, Volume *23*

Supplemental Information

Succinate Dehydrogenase-Regulated Phosphoenolpyruvate Carboxykinase Sustains Copulation Fitness in Aging *C. elegans* Males

Jimmy Goncalves, Yufeng Wan, Xiaoyan Guo, Kyoungsun Rha, Brigitte LeBoeuf, Liusuo Zhang, Kerolayne Estler, and L. René Garcia

Supplemental Figures

Figure S1. Amino acid alignments of *C. elegans* PCK-1 and PCK-2, Related to Figure 1. The red boxes denote positions of amino acids that participate in oxaloacetate and GTP binding and phosphoenolpyruvate formation (Carlson and Holyoak, 2009; Sullivan and Holyoak, 2007).

```
PCK-1   7     SLRNMETDGFQVVTEVVTHKLNHIPIFKGDFASLSPKVQRFVAEKAELMNPAGIYICDGS  66
              SLR +  D F VV EVV  +L H+PI KGDF  L  KVQRF+AEKAELM P GI+ICDGS
PCK-2   20    SLRQISEDAFYVVNEVVMKRLGHVPILKGDFHLLPAKVQRFIAEKAELMRPRGIFICDGS  79

PCK-1   67    QKEYDDIVDKLVERGVLTPLKAYENNYLCRTDPRDVARVESKTWMVTKDKYDSVCHTPDG  126
              Q E D+++DKL+ERG+L+ L+AYENNY+CRTDP+DVARVESKTWMVTK+KYD+V HT +G
PCK-2   80    QHEADELIDKLIERGMLSKLEAYENNYICRTDPKDVARVESKTWMVTKNKYDTVTHTKEG  139

PCK-1   127   VRPIMGQWMSEEQFGVELDSRFPGCMAGRPMYVVPYSMGPIGGPLSKNGIELTDSPYVVL  186
              V PIMG W++ E    ELDSRFPGCMAGR MYV+P+SMGP+GGPLSK GI+LTDS YVVL
PCK-2   140   VEPIMGHWLAPEDLATELDSRFPGCMAGRIMYVIPFSMGPVGGPLSKIGIQLTDSNYVVL  199

PCK-1   187   CMRTMTRMGTKVLEALGDNDFVRCIHSVGLPRPVKQKVINHWPCNPEKVMIAHRPKEREI  246
               MR MTR+   V +ALG+ DFVRCIHSVGLPRPVKQ+VINHWPCNPE+V+IAHRP EREI
PCK-2   200   SMRIMTRVNNDVWDALGNQDFVRCIHSVGLPRPVKQRVINHWPCNPERVLIAHRPPEREI  259

PCK-1   247   WSFGSGYGGNSILGKKCFALRIACNIGRDEGWLAEHMLIMGVTNPEGEEKFIAAAFPSAC  306
              WSFGSGYGGNS+LGKKCFALRIA NI +DEGW+AEHMLIMGVT P G E FIAAAFPSAC
PCK-2   260   WSFGSGYGGNSLLGKKCFALRIASNIAKDEGWMAEHMLIMGVTRPCGREHFIAAAFPSAC  319

PCK-1   307   GKTNLAMLTPTVPGWKVRVVGDDIAWMKFGADGRLYAINPEAGFFGVAPGTSHKTNAMAM  366
              GKTNLAML PT+PGWKVR VGDDIAWMKFG DGRLYAINPEAGFFGVAPGTS+KTN MA+
PCK-2   320   GKTNLAMLEPTLPGWKVRCVGDDIAWMKFGEDGRLYAINPEAGFFGVAPGTSNKTNPMAV  379

PCK-1   367   ESCRANTIFTNVAETADGEYFWEGLEKELKEAKGYTDEQLKHLEITNWLGERWHIGDEGK  426
               + + N+IFTNVAETA+GEYFWEGLE E+ +          K+++IT WLGE+WHIG+ G
PCK-2   380   ATFQKNSIFTNVAETANGEYFWEGLEDEIAD---------KNVDITTWLGEKWHIGEPGV  430

PCK-1   427   AAHPNSRFTAPAKQCPNIHPDWEAPQGVPIDAIVFGGRRPEGVPLVFESFSWEHGILVGA  486
              AAHPNSRF APA QCP IHPDWE+PQGVPI+AI+FGGRRP+GVPL++E+ SWEHG+  G+
PCK-2   431   AAHPNSRFAAPANQCPIIHPDWESPQGVPIEAIIFGGRRPQGVPLIYETNSWEHGVFTGS  490

PCK-1   487   LVKSETTAAAEFTGKNVMHDPMAMRPFMGYNYGKYLEHWIKLGKAPHKAPKIFHVNWFRE  546
               +KSE TAAAEFTGK VMHDPMAMRPFMGYN+GKYL+HW+ L     K PKI+HVNWFR+
PCK-2   491   CLKSEATAAAEFTGKTVMHDPMAMRPFMGYNFGKYLQHWLDLKTDSRKMPKIYHVNWFRK  550

PCK-1   547   TKDHKFLWPGFGDNIRVLDWILRRVAGGEEEIAIETAIGYVPKRGTINLDGLPRIDWNDL  606
                ++KFLWPGFGDNIRV+DWI+RR+  GE+EI +ET IG VP +G+INL+GL  ++W++L
PCK-2   551   DSNNKFLWPGFGDNIRVIDWIIRRL-DGEQEIGVETPIGTVPAKGSINLEGLGEVNWDEL  609

PCK-1   607   MSIPKDYWVEDVDESRHFLDTQVGSDLPQPIRDELDKLEKRVHAL  651
              MS+P DYW +D  E R FLD QVG DLP+P+R E+D  EKRV  L
PCK-2   610   MSVPADYWKQDAQEIRKFLDEQVGEDLPEPVRAEMDAQEKRVQTL  654
```

Figure S2. Amino acid alignments of *C. elegans* PCK-2 and rat PCK-1, Related to Figure 1. The red boxes denote positions of amino acids that participate in oxaloacetate and GTP binding and phosphoenolpyruvate formation.

```
CePCK-2   45   ILKGDFHLLPAKVQRFIAEKAELMRPRGIFICDGSQHEADELIDKLIERGMLSKLEAYEN  104
               +++G    LP +V++F+   A+L +P  I ICDGS+ E   L+  + E G++ KL+ Y+N
RatPCK-1  15   VIQGSLDSLPQEVRKFVEGNAQLCQPEYIHICDGSEEEYGRLLAHMQEEGVIRKLKKYDN  74

CePCK-2   105  NYICRTDPKDVARVESKTWMVTKNKYDTVTHTKEGVEPIMGHWLAPEDLATELDSRFPGC  164
                ++  TDP+DVAR+ESKT ++T+ + DTV   K G +  +G W++ ED     ++RFPGC
RatPCK-1  75   CWLALTDPRDVARIESKTVIITQEQRDTVPIPKSG-QSQLGRWMSEEDFEKAFNARFPGC  133

CePCK-2   165  MAGRIMYVIPFSMGPVGGPLSKIGIQLTDSNYVVLSMRIMTRVNNDVWDALGNQDFVRCI  224
               M GR MYVIPFSMGP+G PL+KIGI+LTDS YVV SMRIMTR+   V +ALG+ +F++C+
RatPCK-1  134  MKGRTMYVIPFSMGPLGSPLAKIGIELTDSPYVVASMRIMTRMGTSVLEALGDGEFIKCL  193

CePCK-2   225  HSVGLPRPVKQRVINHWPCNPERVLIAHRPPEREIWSFGSGYGGNSLLGKKCFALRIASN  284
               HSVG P P+K+ ++N+W CNPE  LIAH P  REI SFGSGYGGNSLLGKKCFALRIAS
RatPCK-1  194  HSVGCPLPLKKPLVNNWACNPELTLIAHLPDRREIISFGSGYGGNSLLGKKCFALRIASR  253

CePCK-2   285  IAKDEGWMAEHMLIMGVTRPCGREHFIAAAFPSACGKTNLAMLEPTLPGWKVRCVGDDIA  344
               +AK+EGW+AEHMLI+G+T P G++ ++AAAFPSACGKTNLAM+ PTLPGWKV CVGDDIA
RatPCK-1  254  LAKEEGWLAEHMLILGITNPEGKKKYLAAAFPSACGKTNLAMMNPTLPGWKVECVGDDIA  313

CePCK-2   345  WMKFGEDGRLYAINPEAGFFGVAPGTSNKTNPMAVATFQKNSIFTNVAETANGEYFWEGL  404
               WMKF   G L AINPE GFFGVAPGTS KTNP A+ T QKN+IFTNVAET++G  +WEG+
RatPCK-1  314  WMKFDAQGNLRAINPENGFFGVAPGTSVKTNPNAIKTIQKNTIFTNVAETSDGGVYWEGI  373

CePCK-2   405  EDEIADKNVDITTWLGEKWHIGEPGVAAHPNSRFAAPANQCPIIHPDWESPQGVPIEAII  464
               ++ +A   V IT+W  ++W   +    AHPNSRF  PA+QCPII P WESP+GVPIE II
RatPCK-1  374  DEPLA-PGVTITSWKNKEWRPQDEEPCAHPNSRFCTPASQCPIIDPAWESPEGVPIEGII  432

CePCK-2   465  FGGRRPQGVPLIYETNSWEHGVFTGSCLKSEATAAAEFTGKTVMHDPMAMRPFMGYNFGK  524
               FGGRRP GVPL+YE  SW+HGVF G+ ++SEATAAAE  GK +MHDP AMRPF GYNFGK
RatPCK-1  433  FGGRRPAGVPLVYEALSWQHGVFVGAAMRSEATAAAEHKGKVIMHDPFAMRPFFGYNFGK  492

CePCK-2   525  YLQHWLDL-KTDSRKMPKIYHVNWFRKDSNNKFLWPGFGDNIRVIDWIIRRLDGEQEIGV  583
               YL HWL +    + K+PKI+HVNWFRKD N KFLWPGFG+N RV++W+  R++GE    +
RatPCK-1  493  YLAHWLSMAHRPAAKLPKIFHVNWFRKDKNGKFLWPGFGENSRVLEWMFGRIEGEDSAKL  552

CePCK-2   584  ETPIGTVPAKGSINLEGLGEVNWDELMSVPADYWKQDAQEIRKFLDEQVGEDLPEPVRAE  643
                TPIG VP + ++NL+GLG+VN +EL  +  ++W+++ +EI K+L++QV  DLP  +  E
RatPCK-1  553  -TPIGYVPKEDALNLKGLGDVNVEELFGISKEFWEKEVEEIDKYLEDQVNADLPYEIERE  611

CePCK-2   644  MDAQEKRVQTL  654
               + A ++R+  +
RatPCK-1  612  LRALKQRISQM  622
```

Figure S3. Amino acid alignments of *C. elegans* PCK-2 and PCK-3, Related to Figure 1. The red boxes denote positions of amino acids that participate in oxaloacetate and GTP binding and phosphoenolpyruvate formation.

```
PCK-2  33    NEVVMKRLGHVPILKGDFHLLPAKVQRFIAEKAELMRPRGIFICDGSQHEADELIDKLI-  91
             N +++   G VP+LKGD   L  +V  F+ +  +LM P  + IC+GS  EA EL D +
PCK-3  33    NTLIVPNFGQVPVLKGDLTWLSPEVLTFLNDCVQLMTPCAVRICNGSVFEAQELRDAIAN  92

PCK-2  92    -----ERGMLSKLE------AYENNYICRTDPKDVARVESKTWMVTKNKYDTVTHTKEGV  140
                  E+ ML +         Y++  +   D  D       +         + + + EG+
PCK-3  93    EFGNEEQQMLDRFHLKMADIGYDDVSVVTKDRLDAD--PGISLSNASASRTSSSGSGEGI  150

PCK-2  141   EPIM--GHWLAPEDLATELDSRFPGCMAGRIMYVIPFSMGPVGGPLSKIGIQLTDSNYVV  198
             E +    H+++ +         F   M+GR MYV+PFSMG +G   + +G+Q+TD   +V
PCK-3  151   ENVRLSSHYMSQKMFDFNKTKLFDCSMSGRTMYVVPFSMGTIGSRRAVVGVQITDDPVLV  210

PCK-2  199   LSMRIMTRVNNDVWDAL-GNQDFVRCIHSVGLPRPVKQRVINHWPC-NPERVLIAHRPPE  256
             L++R   RV +++WD +    +F+RC+H++G+PRP+ ++++   P   P    +  +  +
PCK-3  211   LNLRTTFRVLSNIWDHIAATTNFLRCVHTIGMPRPIIRKIVTPSPVETPVGSFLVLKHDD  270

PCK-2  257   REIWSFGSGYGGNSLLGKKCFALRIASNIAKDEGWMAEHMLIMGVTRPCGREHFIAAAFP  316
             +E+W+ G  +G     GK  F++  AS +   +GW+AE   I+ +T P  +   I   +
PCK-3  271   QEVWAHGHSFGRTPRYGKT-FSVHAASWLGAKQGWLAESAAILAITNP--KNDTIHVCYS  327

PCK-2  317   SACGKTNLAMLEPTLPGWKVRCVGDDIAWMKFGEDGRLYAINPEAGFGVAPGTSNKTNP  376
             S     +L + +   PGWKV  V +   W+ +  DG++Y  +PE              +
PCK-3  328   SLTTIDSLQLSKGLAPGWKVTVVSEKSVWLHW-HDGKIYGFSPE-------------NDE  373

PCK-2  377   MAVATFQKNSIFTNVAETANGEYFWEGLEDEIADKNVDITTWLGEKWHIGEPGVA-AHEN  435
             M      KN    N+      EY       +I   +   T W  +   +G P  A    N
PCK-3  374   MEELGSPKN--LANLLTGVISEY-------QIQSFDPQSTKWASD---VGVPISALIFAN  421

PCK-2  436   SRFAAPANQCPIIHPDWESPQGVPIEAIIFGGRRPQGVPLIYETNSWEHGVFTGSCLKSE  495
              R     +Q P+I       +GV + A I               +S +    +   +K
PCK-3  422   RRH----DQYPLILEANTWEEGVCVAAGI-------------RVSSMKQQKISEESVKES  464

PCK-2  496   ATAAAEFTGKTVMHDPMAMRPFMGYNFGKYLQHWLDL----KTDSRKM-----PKIYHVN  546
                A+    + ++  PM     + ++  KY++HWL++    K+ S        P+I+  N
PCK-3  465   VEGASP--RRILVECPMLRADAINFSIAKYVKHWLEMGVGVKSSSENFENPPPPQIFFTN  522

PCK-2  547   WFRKDSNNKFLWPGFGDNIRVIDWIIRRLDGEQEIG--VETPIGTVPAKGSINLEGLGEV  604
              + ++ + K LWPG  DN R+ ++I  R     ++   + + +G VP      L+
PCK-3  523   LY-QEVDGKPLWPGGVDNARIFEYIYERCANPADLSKTISSGLGIVPK----TLQLSAGT  577

PCK-2  605   NWDELMSVPADYWKQDAQEIRKFLDEQVGEDLP  637
             N   L+ V   +W  +  ++R F + Q+   LP
PCK-3  578   NLAPLLQVDIRFWLTELNKLRAFFNLQMECSLP  610
```

Figure S4. CRISPR/Cas9 engineered YFP-tagged PCK-2 and EGL-2, Related to Figure 1 and 2. (**A**) Cartoon recombination of *pck-2*::YFP C-terminus fusion into the endogenous genomic region of *pck-2* on chromosome I. Black bars depict exons of *pck-2*. Blue bars depict exons of the downstream gene *suro-1*. Grey bars depict introns. Cartoon introns and exons are drawn to relative scale. (**B**) Cartoon recombination of *egl-2*::YFP C-terminus fusion into the endogenous genomic region of *egl-2* on chromosome V. Black bars depict exons of *egl-2*. Grey bars depict introns. Cartoon introns and exons are drawn to relative scale. (**C**) Confocal images of 24 hour adult male tails that express EGL-2::YFP; in the images, anterior region of the male tail is to the left. (**D**) Average fluorescence intensity of a region of interest (ROI) encompassing the protractors on one side of a male. Fluorescence values are in arbitrary units (A.U). Bars and whiskers represent mean and standard deviation. Numbers of males quantified are listed at the bottom of the graph. Each dot represents on the ROI of a single male. P value was determined using the Mann-Whitney test.

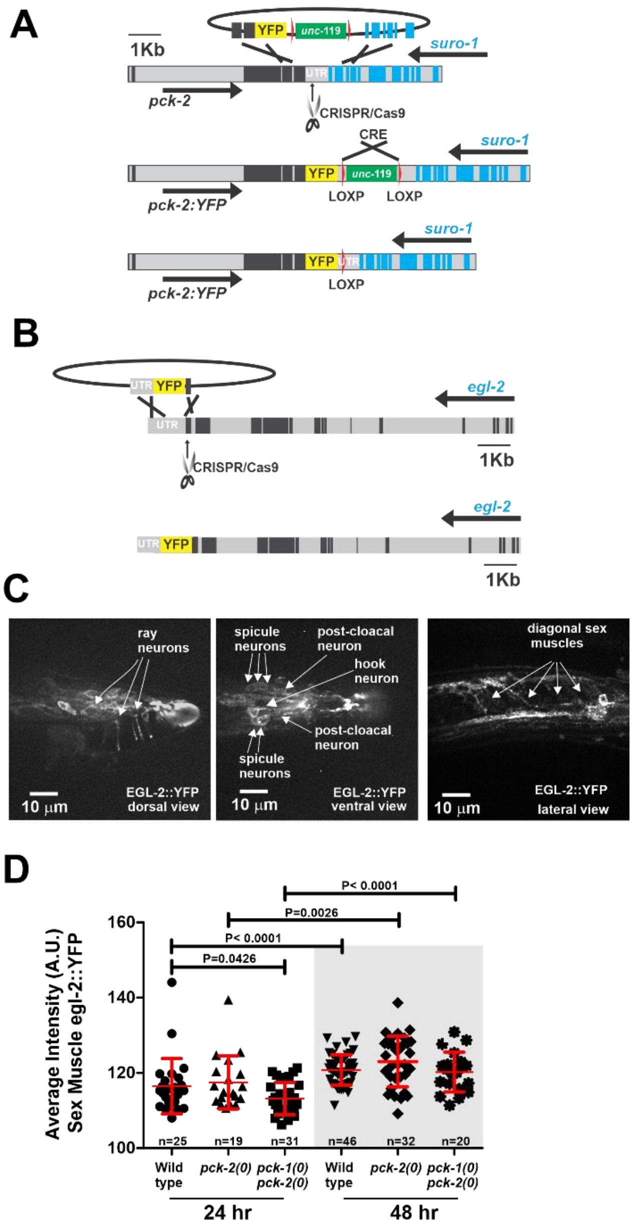

Figure S5. Colocalization analysis of PCK-2::YFP and mitochondria, Related to Figure 1. (**A-C**) Fluorescent micrographs showing cells expressing CFP-tagged mitochondrial protein cyclophilin A (CYN-1::CFP) and PCK-2::YFP of a wild type animal. (**A-B**) Arrows indicate examples of mitochondria where high CYN-1::CFP is seen but PCK-2::YFP is low. White boxes at bottom-right show magnified regions surrounding the arrows. (**D-F**) Fluorescent micrographs showing cells expressing two fluorescent-tagged mitochondrial markers, mitoCFP and PDHA-1::YFP (pyruvate dehydrogenase E1 alpha 1 subunit). (**C and F**) White polygons indicates the regions of interest (ROIs) where both markers are expressed in the cells. (**G-H**) Scatter-plot of the fluorescent intensities of pixels within the ROIs in (**C and F)**. Pearson's correlation coefficient (PCC) and R^2 is calculated as Dunn et al., 2011. Red and green ovals are hypothetical clusters of the pixels. In (**G**) the pixels in the red or the green oval show little correlation between the CFP and YFP, signifying no colocalization, while in (**G**) the pixels in the green oval show strong correlation between the CFP and YFP.

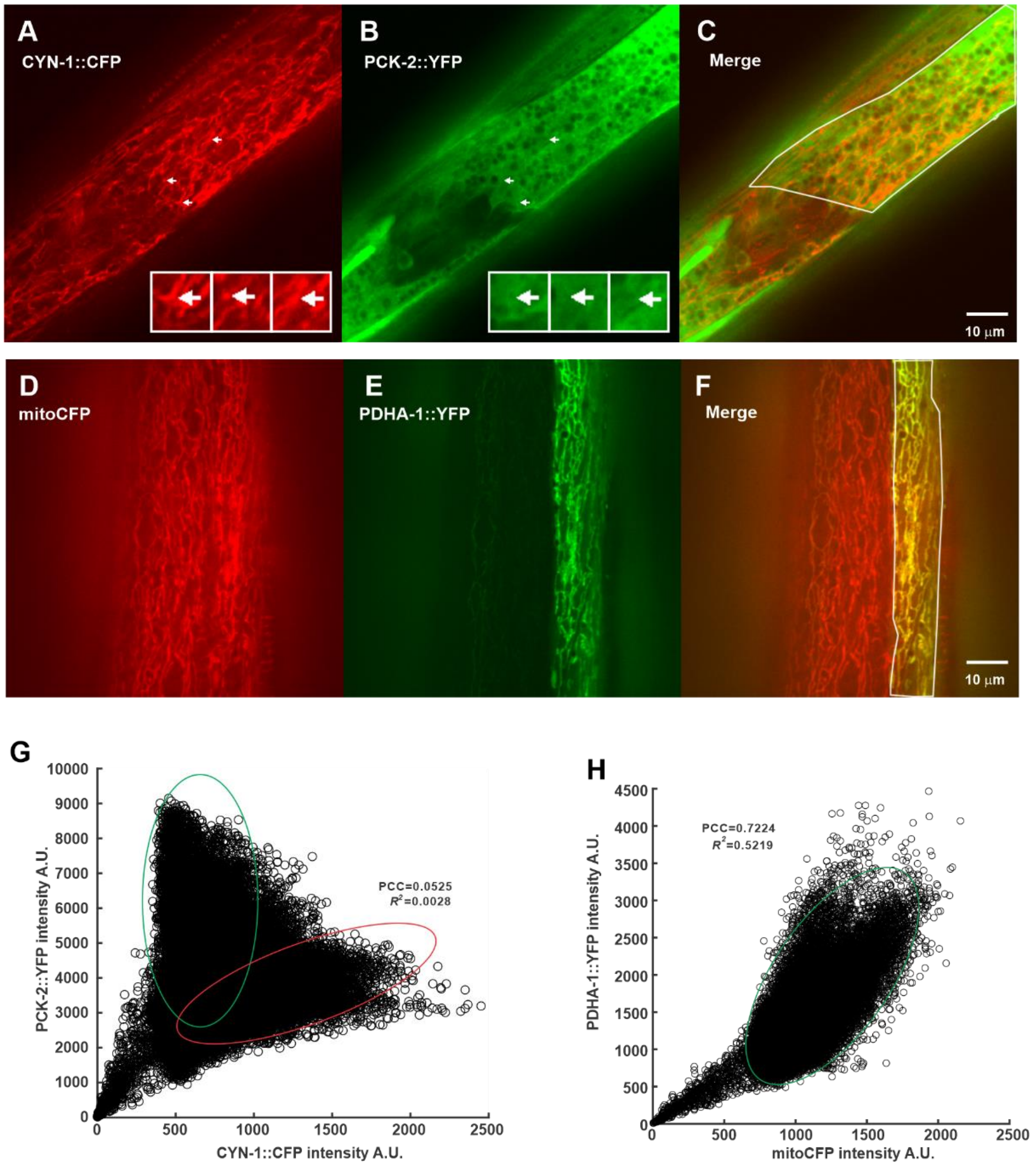

Figure S6. Copulation endurance assay, Related to Figure 3. Grouped column plots of the data shown in Figure 3, depicting the number of females that individual males impregnated over 84 hours. The numbers on the x-axis are the ten individual male designations. The patterned segments within the columns depict the number of females each male impregnated during a specific time interval. Mean and standard deviation represent the number of females that was impregnated by the population of ten males during the 84 hrs assay.

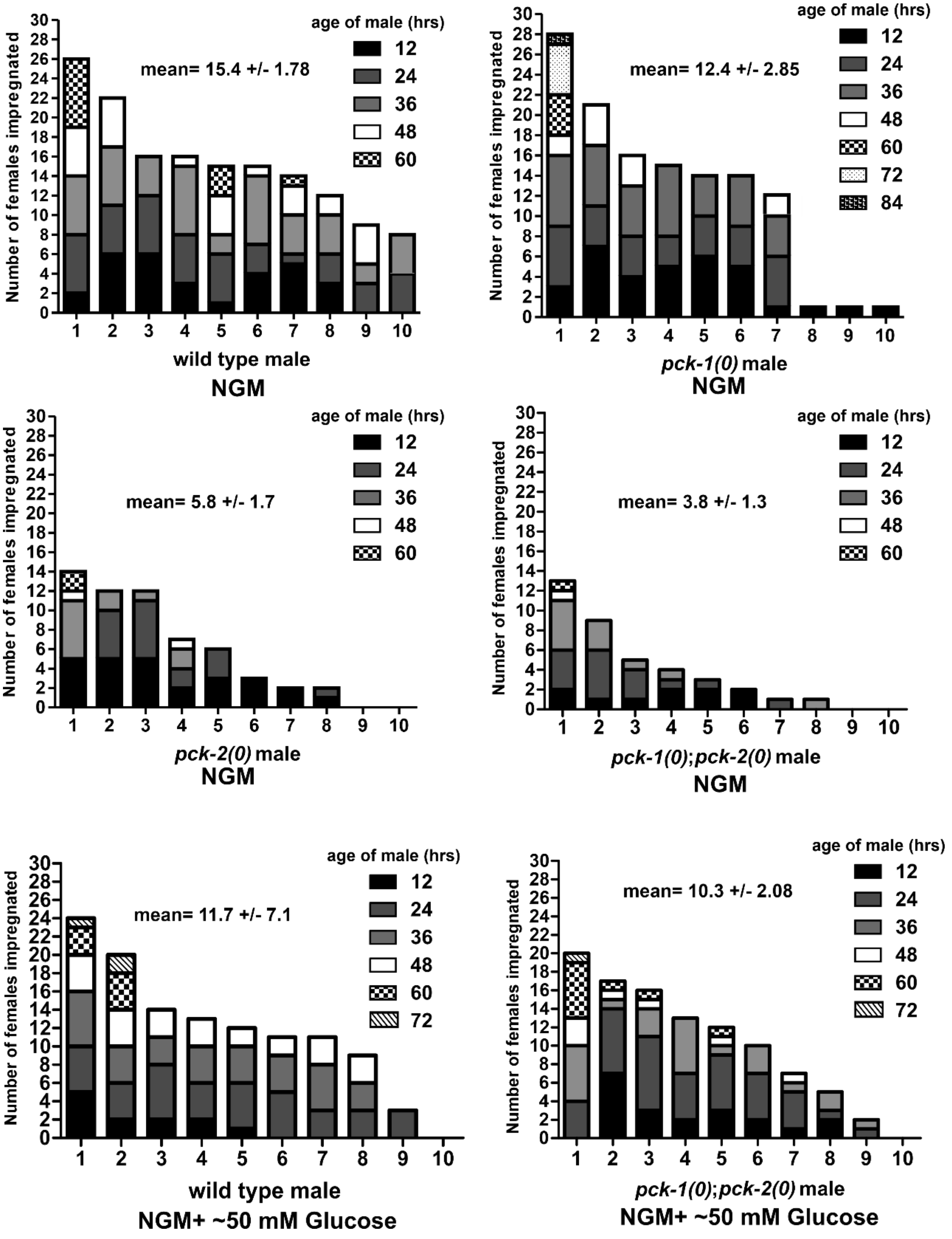

Figure S7. Tissue specific changes in PCK-2::YFP expression with age, Related to Figure 4. Yellow emission fluorescence intensity of muscular, epidermal and intestinal PCK-2::YFP. Fluorescence values are in arbitrary units (A.U). Each symbol represents a single male of the specified age. Bars and whiskers represent mean and standard deviation. P values were determined using the Student's T test.

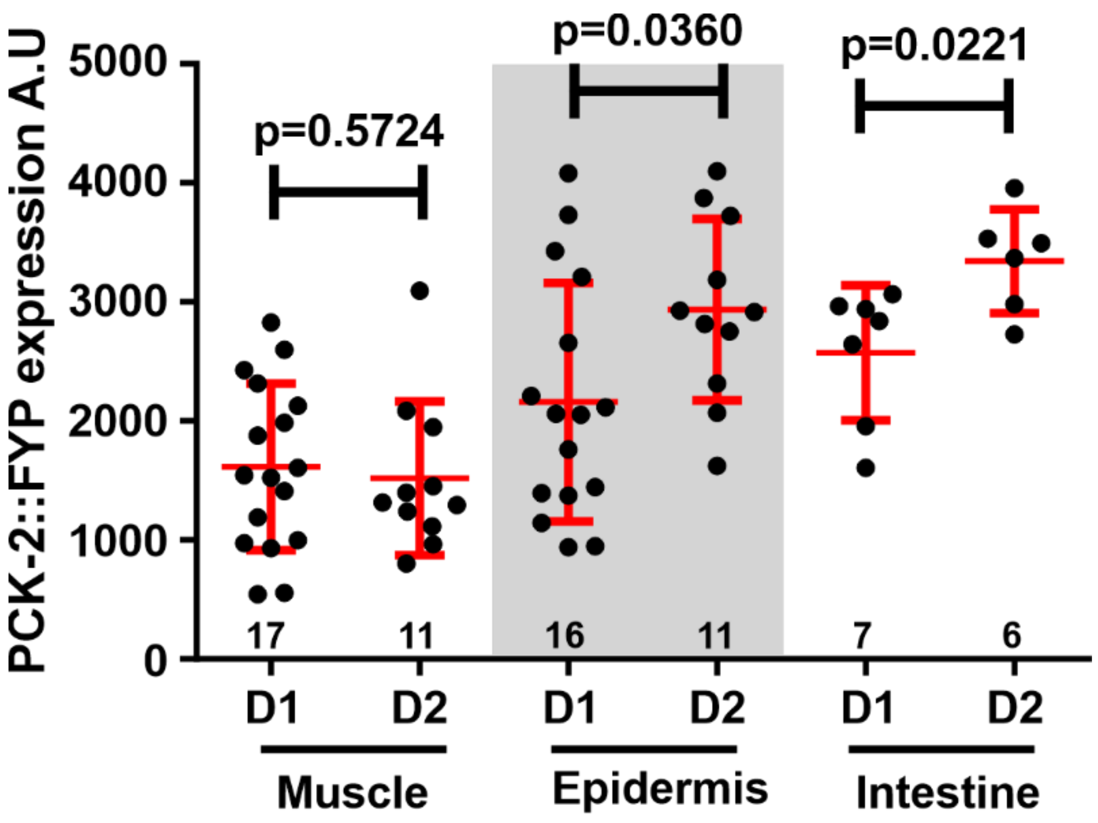

Figure S8. Developmental and physiological phenotypes of the *sdha-1(rg550)* mutant, Related to Figure 5. (**A**) Fluorescent micrographs of mitochondria in the lateral epidermis of a wild type and an *sdha-1* mutant. The *sdha-1* mutant's mitochondria are smaller and more fragmented. The worms contain the integrated transgene *rgIs38* that express both mitochondrial targeted CFP (from the *eft-3* promoter) and also the mitochondrial pyruvate dehydrogenase subunit A fused to YFP (from its native promoter). (**B**) Mitochondria size of wild type and *sdha*-1 mutant using mitochondrial targeted CFP in (**A**). Images were processed to remove background and highlight the mitochondria. Each mitochondrion was then quantified for its size by counting the number of pixels it covers and converting to μm^2 (see Transparent Methods). Columns and whiskers show mean and standard deviation of the mitochondria sizes from 247 and 283 mitochondria of wild type and *sdha-1* mutant, respectively. P-value was calculated using the Mann-Whitney test. (**C**) Oxygen consumption of wild type and *sdha-1(rg550)* hermaphrodites. Oxygen consumption was approximated using scratch built soda lime respirometers (Zhang et al., 2015). The data shown are the mean and standard deviations of three independent trials. Each trial contained 300 day 1 hermaphrodites. Statistical significance was achieved after 4 hours. P value was determined using Student's T test. (**D**) Developmental time course of wild type and *sdha-1(rg550)* hermaphrodites. The developmental morphology of the gonad was used as an approximation for the larval stage of the animal (Kimble and Hirsch, 1979). Some of the developmentally retarded *sdha-1(rg550)* larva with an L2 gonad had P5.p, P6.p and P7.p descendants that had division patterns of an L3 animal. These animals were likely stalled at the L3 stage, but their gonad did not develop past the L2 stage. However by 55 hours, stalled larva progressed to L4 or adult stages. Numbers of hermaphrodites examined at each time point are listed at the bottom of the bars. (**E**) Number of body bends that 20 wild type and 20 *sdha-1(rg550)* 24 hrs adult virgin males produced while thrashing for 1 minute in M9 buffer. The number of animals quantified is 10 per strain. Each symbol represents the number of body bends for 1 male. Bars and whiskers represent mean and standard deviation. P value was determined using the Mann-Whitney test. (**F**) DIC image of a 24 hrs adult wild type male expressing YFP from the *aex-2* promoter. (**F'**) Fluorescence image of the wild type male depicted in (**F**)**;** the remodeled adult anal depressor expresses YFP. The sphincter and intestinal muscles also expresses YFP. (**G**) DIC image of a 24 hrs adult *sdha-1(rg550)* male expressing YFP from the *aex-2* promoter. (**G'**) Fluorescence image of the *sdha-1(rg550)* male depicted in (**G**)**;** the *sdha-1(rg550)* male anal depressor muscle did not remodel and has the appearance of a larval male's anal depressor. (**H**) Survival of adult males grown on paraquat. Live males were determined by whether they thrash in M9 buffer for 10 seconds. Statistical significance was determined by the Mann-Whitney test. Error bars represent the 95% confidence intervals. Total number of animals assayed per strain is noted at the bottom of the column; the % of surviving males is listed at the top of the column. (**I**) Number of eggs laid by a 24 hrs adult wild type and *sdha-1(rg550)* hermaphrodite. The number of animals quantified is 15 per strain. Each symbol represents the number of eggs laid for 1 hermaphrodite. Bars and whiskers represent mean and standard deviation. P-value was calculated using the Mann-Whitney test.

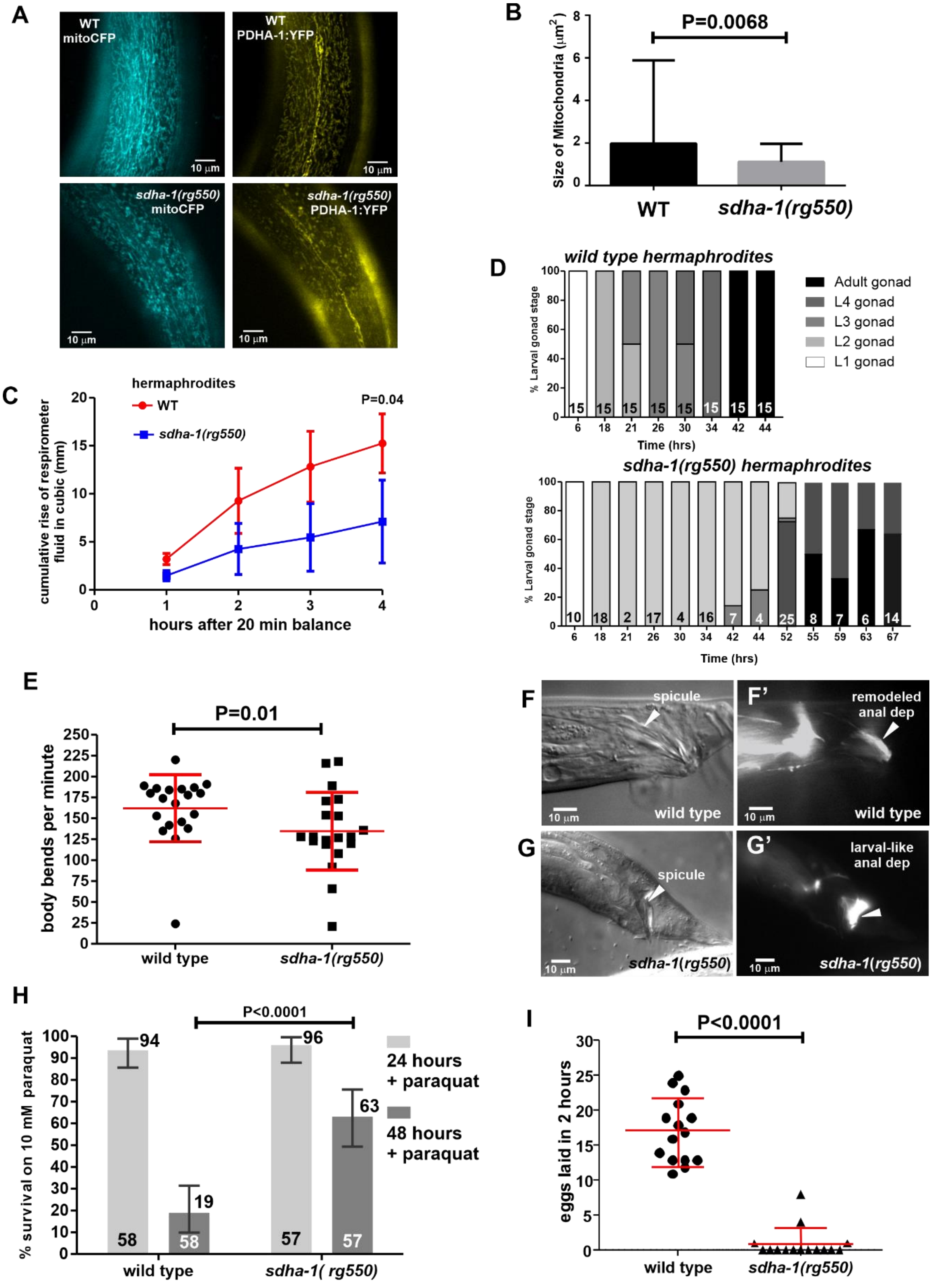
A
WT mitoCFP
WT PDHA-1:YFP
sdha-1(rg550) mitoCFP
sdha-1(rg550) PDHA-1:YFP
10 µm
B
P=0.0068
Size of Mitochondria (µm²)
WT
sdha-1(rg550)
C
hermaphrodites
WT
sdha-1(rg550)
P=0.04
cumulative rise of respirometer fluid in cubic (mm)
hours after 20 min balance
D
wild type hermaphrodites
sdha-1(rg550) hermaphrodites
% Larval gonad stage
Time (hrs)
Adult gonad
L4 gonad
L3 gonad
L2 gonad
L1 gonad
E
P=0.01
body bends per minute
wild type
sdha-1(rg550)
F
F'
spicule
remodeled anal dep
wild type
G
G'
larval-like anal dep
sdha-1(rg550)
H
P<0.0001
% survival on 10 mM paraquat
24 hours + paraquat
48 hours + paraquat
wild type
sdha-1(rg550)
I
P<0.0001
eggs laid in 2 hours
wild type
sdha-1(rg550)

Figure S9. Glycogen and neutral lipid contents, Related to Figure 5. (**A**) Lugo's staining of the isthmus region of pharynx of 24 hrs adult wild type, *pck-2(0)*, *sdha-1(rg550)*, *pck-2(0); sdha-1(rg550)* hermaphrodites and males. Red dotted polygon outlines the isthmus region that is quantified. (**B**) Quantification of glycogen staining of animals in (**A**). Numbers of animals quantified are indicated in the brackets beside the strain names. Bars and whiskers show mean and standard deviation. P values were calculated by One-way ANOVA, Bonferroni's multiple comparison test. (**C**) Nile red staining of fed and starved 24 hour wild type, *pck-2(0)*, *sdha-1(rg550)* and *pck-2(0)*; *sdha-1(rg550)* males. The y-axis represents the average pixel intensity within a series of rectangular ROI drawn over the Nile Red-stained intestine. Bars and whiskers show mean and standard deviation. P values were determined using Student's T test. Numbers of animals quantified is listed at the bottom (top for WT starved) of the graph.

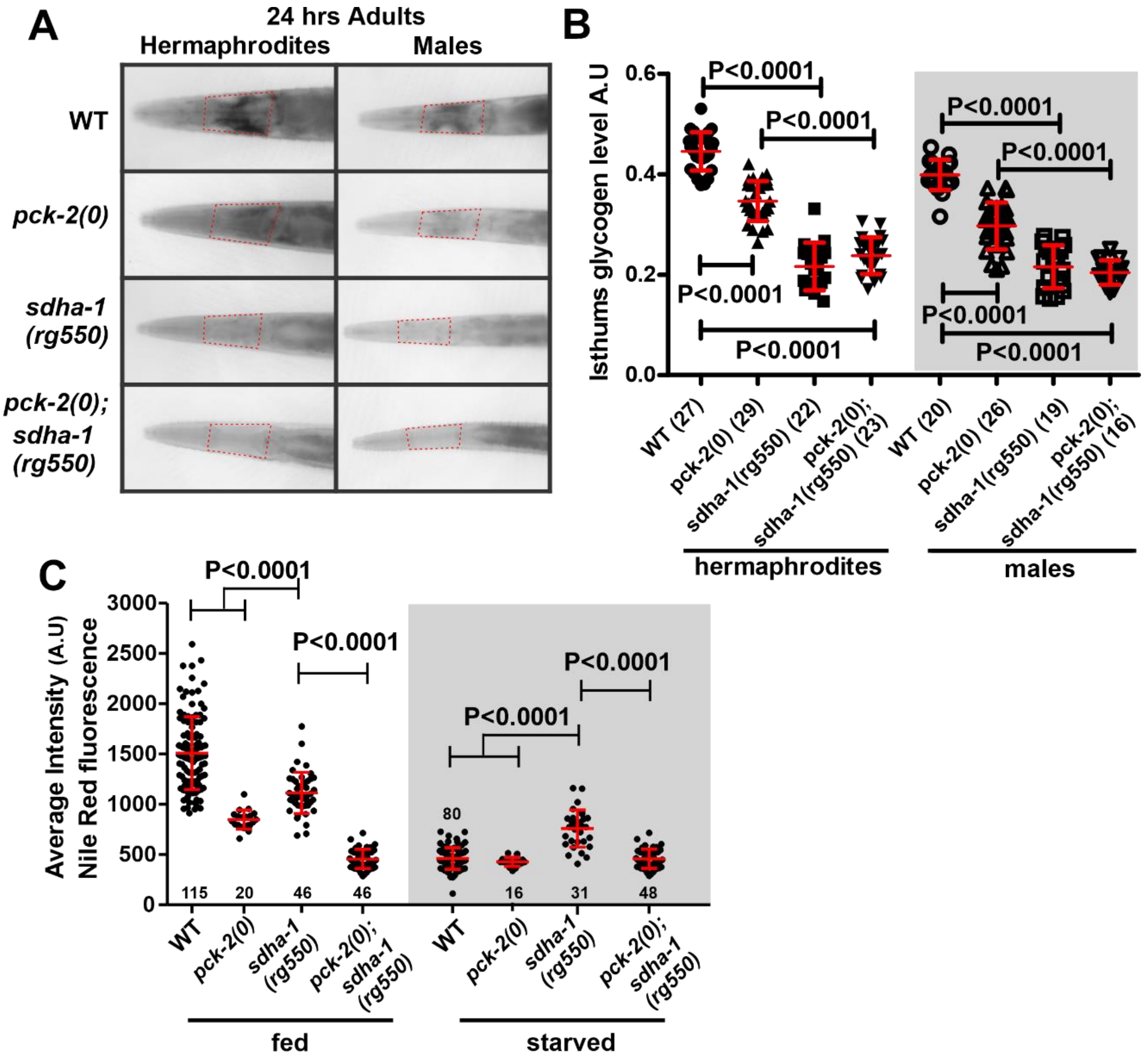

Transparent Methods

Strains

All animals, including the wild type, contain the *him-5(e1490)* allele (Hodgkin et al., 1979) and were grown at 20°C on NGM agar plates containing *E. coli* OP50 (Brenner, 1974). Additional strains used in the study contained the following alleles: *pck-2(ok2586)* (generated by the *C. elegans* Gene Knockout Consortium), *pck-2(rg551), pck-2(rg800, rg551)* (this work) on linkage group (LG) I*; let-23(sy1)* (Aroian and Sternberg, 1991) on LG II; *pck-1(ok2098)* (generated by the *C. elegans* Gene Knockout Consortium), *pha-1(e2123)* (Schnabel and Schnabel, 1990), *mev-1(kn1)* (Ishii et al., 1990) on LG III; *fog-2(q71)* (Schedl and Kimble, 1988), and *egl-2(rg444)* (this work) on LG V.

Potency assay, spicule protraction assay, copulation fitness assay and copulation endurance assay.

The potency assay was conducted as previously described (Guo et al., 2012). In groups of 10 to 15 animals, L4 males (at the developmental stage where the tail spike has retracted) from non-crowded cultures were separated from their hermaphrodite siblings. Every day, males were transferred to fresh NGM-OP50 plates to minimize their exposure to accumulated deleterious male secretions (Shi et al., 2017). *pha-1(e2123)* hermaphrodites were used as mating partners (Schnabel and Schnabel, 1990). *pha-1* cultures were propagated at 15°C (permissive temperature), but 24 hrs before the assay, L4 *pha-1* hermaphrodites were isolated from their siblings and cultured at 20°C (non-permissive temperature). To conduct the potency assay, one male of different ages (newly adult molted, 24 hour (hr) or 48 hr adult) was paired with one 24 hr adult *pha-1* hermaphrodite on a NGM plate containing a 5 mm diameter lawn of OP50. Matings were done at 20°C. Five days later, the mating was scored successful if the plate contains cross-progeny. *pha-1* embryos and L1 larva die at 20°C, but cross-progeny, which are heterozygous for *pha-1,* are viable and reproductive.

The spicule protraction assay was conducted as previously described (Garcia and Sternberg, 2003). Late L4 males (at a developmental stage close to molt) were isolated from hermaphrodites and kept together in groups of 20 to 25 males. In some trials, a group of 20 males was also incubated with five late L4 wild-type or *let-23(sy1)* vulvaless hermaphrodites. ~24 to 26 hours (hrs) later, using a stereo dissecting microscope at 56X magnification, males were scored as have the protraction constitutive phenotype (Prc) if they had one or both copulatory spicules dangling out their cloaca.

The copulation fitness assay was conducted as previously described (LeBoeuf et al., 2011). Briefly, L4 males from non-crowded cultures were separated from their hermaphrodite siblings. A day 1 virgin *fog-2(q71)* mutant female was used as the males' mating partner. *fog-2* mutant females do not make sperm and requires male impregnation to produce progeny (Schedl and Kimble, 1988). One 24 hr adult male containing a genetically-integrated fluorescent marker and one unmarked 24 hr adult male were paired with the virgin *fog-2* mutant female on a 5 mm diameter OP50 lawn. Multiple copulations were periodically monitored every one to two minutes until one of the males was observed to transfer sperm. For the males used in this study, the mating competition lasts between 5 to 20 minutes. After insemination, both males were removed from the female. The next day, the all-or-none fluorescence status of the L1 progeny population was used to determine the paternity of the competing males. If the female produced a mixture of fluorescent and non-fluorescent progeny, then that competition trial was not counted, since both males in the competition mated with the female during a period that the copulation was not being observed.

The copulation endurance assay measures how many different partners a single male can impregnate. The assay is modified from Hodgkin and Doniach 1997 (Hodgkin and Doniach, 1997). Plates containing overnight lawns of OP50 were exposed to 1 Joule of UV, using a Stratagene UV-Stratalinker 1800. The killed bacteria were washed off the plates, concentrated and then spotted on NGM plates without or with D-glucose (Sigma) at a final concentration of 56 mM (~1% glucose). Mid-L3 males were then separated from hermaphrodites and grown on plates +/- glucose. One newly molted adult male and ten newly molted virgin *fog-2* mutant females were placed on a 5 mm diameter lawn of UV- killed OP50 (+/- glucose). Every 12 hrs for 96 hrs, females that contained eggs in their uterus were counted and then

removed from the assay. The male and remaining un-mated females were then moved to a fresh 5 mm diameter UV-killed OP50 lawn and new age-matched virgin *fog-2* mutant females (virgin females were always the same age as the male) were added to replace the mated ones; thus the bacterial lawn always contain ten females.

Data analyses.

Statistical tests were performed using Graph Pad Prism 5.04 for Windows (Graphpad Software, San Diego, CA USA). Confidence intervals for the competition assay were calculated using the formula: CI= $p \pm Z_{\alpha/2}\sqrt{(p(1-p)/n)}$, where p is the fraction of males that won the competition, n is the number of independent competitions, and $Z_{\alpha/2}$ is set at the 95% level, which is 1.96.

Construction of plasmids and transgenic strains

pck-1 promoter and expression plasmid and transgenic strains.

To generate the promoter for *pck-1*, a 2888 base pair (bp) DNA region between the upstream gene *spt-4* and the first predicted *pck-1* exon was PCR-amplified using the primers Pck-1attb1: GGGGACAAGTTTGTACAAAAAAGCAGGCTGGTCGATTTTGAAGATTTTTCAGACCCAAAC and Pck-1attb2: GGGGACCACTTTGTACAAGAAAGCTGGGTACACTCGTTTGCTATGGTTCTGGAATAGGTT. The PCR product was recombined into the gateway entry vector pDG15 (Reiner et al., 2006) using BP clonase (Invitrogen) to generate pXG47. Using LR clonase (Invitrogen), the *pck-1* promoter from pXG47 was then recombined with the DsRed1-E5 -containing Gateway destination plasmid pLR186 (LeBoeuf et al., 2011) to generate the plasmid pXG55. 50 ng/µl pXG55 and 150 ng/µl pUC18 were then micro-injected into N2. After a stable transgenic line was obtained, the extrachromosomal array was integrated into the genome using trimethylpsoralen and UV to promote DNA breakage and repair (Anderson, 1995). The extrachromosomal array integrated into chromosome I to generate the transgenic allele *rgIs29*.

To construct *pck-1*-containing plasmids, the *pck-1* spliced variant cDNA W05G11.6a.1 (http://www.wormbase.org/db/get?name= WBGene00021043;class=Gene) was purchased as a G-block from IDT technologies. Using In-Fusion HD-cloning (Takara Bio), the cDNA was fused at the 3'end to YFP by cloning into the gateway plasmid pGW322YFP, to generate the plasmid pLR385 (Reiner et al., 2006). LR clonase was used to recombine the *unc-103*A promoter (using the gateway entry plasmid pLR36 (Reiner et al., 2006)) and the *unc-17* promoter (using the Gateway entry plasmid pBL228 (LeBoeuf et al., 2014)) into pLR385 to generate the plasmids pLR386 and pLR387, respectively. The *pck-1* promoter from pXG47 was recombined with pLR385 using LR clonase to generate pLR388. A 1882 bp genomic region from *pck-1*, which contains the first three introns, was PCR- amplified from N2 DNA using the primers: ForPck1seq2: AGCTCGTCGAGCGTGGTGTCCTTACand Revpck1seq5: GAGGTTGGTCTTTCCGCAGGCGGAT. This region was recombined into pLR388 using In-Fusion HD-cloning to generate pLR389. Injection mixes, containing 20 ng/µl of pLR386, or 10 ng/µl pLR387 or 20 ng/µl of pLR388 and up to 180 ng/µl of pUC18 filler DNA, were injected into *pck-1(ok2098); pck-2(ok2586); him-5(e1490)* hermaphrodites. Stable transgenic lines that heritably transmit YFP fluorescence were kept to generate the extrachromosomal arrays: *rgEx842*[$P_{pck\text{-}1}$:*pck-1*::YFP], *rgEx847*[$P_{unc\text{-}103A}$:*pck-1*::YFP] and *rgEx848*[$P_{unc\text{-}17}$:*pck-1*::YFP]. To over-expresses *pck-1*:YFP, an injection mix containing 50 ng/µl of pLR389, 50 ng/µl of the *pha-1(+)* rescuing plasmid pBX1 (Granato et al., 1994) and 100 ng/µl of pUC18 was injected into *pck-2(rg551)*;*pha-1(e2123); him-5(e1490)*. A stable transgenic line was kept to generate the extrachromosomal array: *rgEx878*[$P_{pck\text{-}1}$:*pck-1*::YFP(*OE*); *pha-1(+)*]. A control injection, just containing 50 ng/µl pBX1 and 150 ng/ µl pUC18, was injected into *pck-2(rg551)*;*pha-1(e2123); him-5(e1490)* to generate the array *rgEx877* [*pha-1(+)*].

pck-2 CRISPR/Cas9 plasmids, expression plasmids and transgenic strains.

To generate the *pck-2* CRISPR/Cas9 guide RNA plasmid pXG67, the 19 bp guide RNA sequence to the *pck-2* 3' untranslated region (UTR) (5' TTTTTCGCGCATTATTTCA 3'), was added to the CRISPR/Cas9 guide RNA/ enzyme plasmid pDD162 (Dickinson et al., 2013) using PCR and the primers, pck-2sgRNAFOR: TTTTTCGCGCATTATTTCAGTTTTAGAGCTAGAAATAGCAAGT and sgRNA(universal)REV: CAAGACATCTCGCAATAGG. To generate the CRISPR/Cas9 guide RNA plasmid

pYW52, the 19 bp guide RNA sequence to the *pck-2* third exon region (5'AACGGAGAGTACTTCTGGG 3'), was added to the CRISPR/Cas9 guide RNA/ enzyme plasmid pDD162 using PCR and the primers, pck-23rdexgRNA: AACGGAGAGTACTTCTGGGGTTTTAGAGCTAGAAATAGCAAGT sgRNA(universal)REV: CAAGACATCTCGCAATAGG. To generate the *egl-2* CRISPR/Cas9 guide RNA plasmid pJG3, the 19 bp guide RNA sequence to the *egl-2* 3' untranslated region (UTR) (5' CGAGCTTGAAGCCGGCGAG 3') was added to the CRISPR/Cas9 guide RNA/ enzyme plasmid pDD162 using PCR and the primers FEgl2cas9: CGAGCTTGAAGCCGGCGAGGTTTTAGAGCTAGAAATAGCAAGT and sgRNA(universal)REV: CAAGACATCTCGCAATAGG.

The *pck-2* repair template used for CRISPR/Cas9-mediated recombination was generated by PCR-amplifying from genomic DNA a ~ 3.7 kilo base pair (kb) fragment containing the terminal end of the *pck-2* coding region, UTR and part of the downstream gene *suro-1*. This fragment was amplified using the primers pck-25'armfor: CACCAAGAACAAGTATGACACTGTCACAC and pck-23'armrev: TTCAGTGAGCCTGAAACAAGAGCTGTTCG. The fragment was then inserted into pCRXLTOPO to generate pXG66. We amplified YFP from a stock YFP-containing plasmid (pGW322YFP) using the primers pck-2a::gfpFOR: GAGAGTCCAGACATTGATGAGTAAAGGAGAAGAACTTTTC and pck-2a::gfpREV: GGTATAATAACTATTTGTATAGTTCATCCATGC. We also amplified *C. briggsae* (*Cb*) unc-119 flanked with *loxP* sites using the primers *unc-119(cb)*FOR: AATTTGATTTTATAACTTCGTATAGCATACATTATACGAAGTTATCTTTGAGCCAATTTATCCAAG and unc-119(cb)REV: TCATCAAACGTCACTGAATAACTTCGTATAATGTATGCTATACGAAGTTATCCTAGTTCTAGACATTCTCTAATG. Using the In-Fusion HD-cloning, we translationally fused YFP to the last codon of *pck-2* in the plasmid pXG66. Flanked *loxP-Cb* unc-119 was then added to the *pck-2* 3'UTR to make the final plasmid pXG68. Plasmids were sequenced and repaired for mutations that arose during the construction.

Plasmids used for conducting tissue-specific rescue of *pck-2*(0) were constructed as follows. Using inverse PCR, we removed *unc-119*(*Cb*) from the plasmid pXG68 with flanking primers. The resulting plasmid, pLR366 still contains a *loxP* site in the 3' UTR. ~3.7 kb *pck-2* DNA encompassing the third exon, third intron and part of the fourth exon was PCR-amplified using the primers AMP*pck2*: GTGTGCTGGAATTCGCCCTTAGCGTCTTGGACATGTTCCGATTCTCAAG and reverseAMP*pck2*: CCTTGGTGTGTGTGACAGTGTCATACTTGTTCTTGGTG. In-Fusion HD-cloning was used to fuse this fragment of DNA with *pck-2* sequences in the plasmid pLR366 to generate pLR369. A second *loxP* site was introduced into the third intron. The large second intron of *pck-2* contains multiple repeat sequences making it unstable, therefore we directly purchased the first exon, first intron and second exon sequences of *pck-2* as a G-block from IDT technologies and using In-Fusion HD-cloning, fused the fragment into pLR369, to generate pLR373. The Invitrogen Gateway ATTR cassette B was cloned into the *Sal*I site, immediately upstream of the *pck-2*'s first ATG to generate pLR374.

pLR374 is the *pck-2*::YFP expression vector that different tissue promoters were recombined with using Invitrogen LR clonase . For intestinal expression, the gateway entry plasmid pBL63 (LeBoeuf et al., 2007) was used to recombine in the *gtl-1* promoter (Teramoto et al., 2005) to generate pLR379. For body wall muscle, sex muscle, intestinal and anal depressor muscle expression, the gateway entry plasmid pLR36 (Reiner et al., 2006) was used to recombine in the *unc-103*A promoter (Reiner et al., 2006) to generate the plasmid pLR382. For sex muscle expression and seven pairs of head neurons, the gateway entry plasmid pBL458 (LeBoeuf and Garcia, 2017) was used to recombine the small *unc-103*E promoter (Reiner et al., 2006) to generate pLR380. For epidermal expression, the gateway entry plasmid pXG76 was used to recombine the *dpy-7* promoter (Gilleard et al., 1997) to generate pLR381. For general worm over-expression, the gateway entry plasmid pYW34 was used to recombine the *eft-3* (also known as glp-3 and *eef-1A*) promoter (Newbury et al., 2007) to generate pLR375.

Injection mixes, containing 20 ng/µl of pLR379, pLR382, pLR380 or pLR381 and up to 180 ng/µl of pUC18 filler DNA, were injected into the germline of *pck-1(ok2098); pck-2(ok2586); him-5(e1490)* hermaphrodites. Stable transgenic lines that heritably transmit YFP fluorescence were kept to generate the extrachromosomal arrays: *rgEx833*[$P_{gtl\text{-}1}$:*pck-2*::YFP], *rgEx879*[$P_{unc\text{-}103A}$:*pck-2*::YFP] , *rgEx834*[$P_{unc\text{-}103E}$:*pck-2*::YFP] and *rgEx837*[$P_{gtl\text{-}1}$:*pck-2*::YFP].

mitoCFP, cyn-1, pdha-1 and sdha-1 promoter plasmids, expression plasmids and transgenic strains.

To generate mitochondrial targeted CFP vector, the F22B7.9 promoter was removed from the Andy Fire mitochondria-targeted CFP vector pPD135.41 (Addgene plasmid repository). T4 ligase cloning was used to add the Gateway cassette C.1 (Invitrogen) in front of the mitochondrial targeted protein to generate the Gateway entry vector pLR339. Using LR clonase, the *eft-3* promoter from the plasmid pYW34 was recombined with pLR339 to generate the plasmid pJG52. To fused the pyruvate dhydrogenase subunit A (*pdha-1*)promoter and gene to YFP, A 2.4 kb region that encompasses the promoter and coding exons was PCR- amplified from N2 genomic DNA using the primers FORPDHA1Prom: GGGGACAAGTTTGTACAAAAAAGCAGGCTGCAACTCATTGGTAGCAAGGCCTCCGAGTT and REVPDHA1: GGGGACCACTTTGTACAAGAAAGCTGGGTAGCTCTTCCAATAGACTTCAGTACATCAGCA. Using BP clonase, the PCR fragment was recombined into the Gateway entry vector pDONR221 to generate pJG34. The *pdha-1* fragment in pJG34 was then recombined into the YFP-containing gateway destination vector pGW322YFP using LR clonase to generate pJG35. An injection mix containing 20 ng/µl of pJG52 and 20 ng/µl of pJG35 and 160 ng/µl of pUC18 was injected into the germline of N2 hermaphrodites. After a stable transgenic line was obtained, the extrachromosomal array was integrated into chromosome V to generate *rgIs38*[$P_{eft\text{-}3}$:mito-targeted CFP; $P_{pdha\text{-}1}$:*pdha-1*::YFP].

To generate cyclophylin A *cyn-1* expression plasmids, the *cyn-1* genomic sequence was PCR amplified from N2 DNA using the primers GW322Forcyn-1: TTGAGGGTACCGGTAGAAAAAATGAAATTTCTACTCCGTGCCTCCTCA and RevCyn-1CFP: AAGTTCTTCTCCTTTACTCATCTCGCTCTTCAACTCTCCGCAATCGGC. Using In-Fusion HD-cloning, the 639 bp fragment was fused at the 3'end to CFP by cloning into the gateway plasmid pGW77CFP, creating the Gateway destination vector plasmid pSR20. LR clonase was used to introduce the heat shock promoter (*hsp-16*) from the plasmid pBL172 in front of *cyn-1* of pSR20 to generate pSR21. An injection mix containing 20 ng/µl of pSR21 and 50 ng/µl of pBX1 and 130 ng/µl of pUC18 was injected into the germline of *pha-1*(e2123); *him-5(e1490); lite-1(ce314)* to generate the array *rgEX842*[$P_{hsp\text{-}16}$:*cyn-1*::CFP; *pha-1(+)*].

To generate *sdha-1* expression plasmids, the *sdha-1* genomic sequence was PCR-amplified from N2 DNA using the primers Sdha-1-cfpF: GTACCGGTAGAAAAAATGCTCCGAGCCGCCAGCAAC and Sdha-1-cfpR: TTCTCCTTTACTCATATAGGAGCGGACCTTTGGTGGCA. Using In-Fusion HD-cloning, the 2788 bp fragment was fused at the 3'end to CFP by cloning into the gateway plasmid pGW77CFP, creating the Gateway destination vector plasmid pXG110. LR clonase was used to introduce the heat shock promoter (*hsp-16*) from the plasmid pBL172(Banerjee et al., 2015) in front of *sdha-1* of pXG110 to generate pXG111. An injection mix containing 20 ng/µl of pXG111 and 50 ng/µl of pBX1 and 170 ng/µl of pUC18 was injected into the germline of *pha-1(e2123)*;*him-5(e1490)* to generate the array *rgEx773*[$P_{hsp\text{-}16}$:*sdha-1(+)*::CFP; *pha-1(+)*]. The array was then crossed into *pck-2(rg551)*:YFP; *pha-1(e2123)*; *him-5(e1490); sdha-1(rg550)*.

To generate the *sdha-1* CRISPR/Cas9 guide RNA plasmid pXG128, the 19 bp guide RNA sequence to the 4th exon of *sdha-1* (5' TGGAGAACGGAGTCTGTGT 3'), was added to the CRISPR/Cas9 guide RNA/ enzyme plasmid pDD162 using PCR and the primers, sdha1sgRNAFOR: ATGGAGAACGGAGTCTGTGTGTTTTAGAGCTAGAAATAGCAAGT and sgRNA(universal)REV: CAAGACATCTCGCAATAGG.

The CFP fusion to the C- terminus of SDHA-1 rescued the elevated PCK-2::YFP levels in *sdha-1(rg550)* animals, but did not rescue any other defects; we reasoned that the CFP likely interfered with Complex II function. Additional constructs were made to uncouple CFP from *sdha-1* and also to add *loxP* sites in front of *sdha-1* and after CFP, to be used in future studies. Building on the plasmid pXG110, inverse PCR cloning and the primers UpForloxPsdha1: GCATACATTATACGAAGTTATCCGGTAGAAAAAATGCTCCGAGCCGCCAGC and RevLoxPCFP: TATACGAAGTTATTACCCTCAAGGGTCCTCCTGAAAATG were used to add a *loxP* site in front of the ATG of *sdha-1*. Inverse PCR cloning and the primers: DwnFsdha1oxPcfp: GCATACATTATACGAAGTTATCATTCGTAGAATTCCAACTGAGCGCCGGTC and

DwnRevsdha1oxPcfp: TATACGAAGTTATCTATTTGTATAGTTCATCCATGCCATGTGT were also added downstream of CFP. The primers Forsdha1STOPSL2: CTGGGTGCCACCAAAGGTCCGCTCCTATTAGCCCCAACAGAGTTGT and RevSL2CFP: GAATTGGGACAACTCCAGTGAAAAGTTCTTCTCCTTTACTCATCGGTACCCTCAAGGG were used to add a stop codon to *sdha-1* and PCR-amplify the splice-leader sequence SL2 from one of our existing plasmids pLR279 (Correa et al., 2012). Using In-Fusion HD-cloning, the stop codon-SL2 region was inserted between *sdha-1* and CFP to make the plasmid pLR363.

A 4000 bp promoter region upstream of *sdha-1* was PCR-amplified from N2 DNA using the primers ForATTBsdha-1Prom4kb: GGGGACAAGTTTGTACAAAAAAGCAGGCTACAGTGGGCGCACAGGTGGTAACGAGAAACA and RevATTBsdha-1Prom4kb: GGGGACCACTTTGTACAAGAAAGCTGGGTTTTTCCCTGAAAATCGATAAACGTGATATAC. Using BP clonase, the PCR fragment was then inserted into the Gateway entry vector pDG15 to generate the plasmid pJG43. Using primers that reduced the size of the promoter region, the *sdha-1* promoter was shortened to 500 bp to make the plasmid pLR377. Using LR clonase, the 500 bp *sdha-1* (pLR377) and the *unc-103A* (pLR36) promoters were then recombined into pLR363 to generate the plasmids pJG47 and pLR376, respectively. 200 ng/µl injection mixes containing 20 ng/µl of pJG47 or 20 ng/µl of pLR376, and 50 ng/µl of pBX1 and 130 ng/µl of pUC18 were injected into the germline of *pha-1(e2123)*; *him-5(e1490); rgIs2* to generate the extrachromosomal arrays *rgEx819*[P_{sdha-1}:*sdha-1*::SL2:::CFP; P_{ges-1}:mCherry; *pha-1(+)*] and *rgEx836*[$P_{unc-103A}$:*sdha-1*::SL2:::CFP; *pha-1(+)*], respectively. *rgIs2* is an integration of an extrachromosomal array that contains P_{slo-1}: Timer on LG X. The arrays were crossed into *pha-1(e2123)*;*him-5(e1490); sdha-1(rg550). sdha-1(rg550)* is on LGX. In the possibility that the transgenic arrays might rescue the *sdha-1(rg550)* phenotype, *rgIs2* was used as a repulsive fluorescent marker to homozygous the *sdha-1(rg500)* allele.

***egl-2* CRISPR/Cas9 plasmids.**

The *egl-2* repair template, used for CRISPR/Cas9-mediated recombination, was generated by PCR-amplifying from genomic DNA a 900 bp fragment containing the last two exons of *egl-2* coding region and the 3' UTR. This fragment was generated using the primers ATTB1Foregl2cterm: GGGGACAAGTTTGTACAAAAAAGCAGGCTCAGTCAACGATGCCAGTTGGCTCATTTTCTGCGCACCAAGAACAAGTATGACACTGTCACAC and ATTB2Revegl2cterm: GGGGACCACTTTGTACAAGAAAGCTGGGTATGCCCACTTTCATCGCTACTTGCCGACTGA. The fragment was then inserted into pDONR221 (Invitrogen) using BP clonase to generate pJG1. We amplified YFP from a stock YFP-containing plasmid (pGW322YFP) using the primers Fegl-YFP-cterm: CGCGAGCTTGAAATGAGTAAAGGAGAAGAACTTTTCACTGGAG and Regl-cterm-yfp: CCACTCGCCGGCTTTGTATAGTTCATCCATGCCATGTGTAATCTGG. Using In-Fusion HD-cloning, YFP was translationally fused to the last codon of *egl-2* in the plasmid pJG1, to make the plasmid pJG2.

Construction of Paex-2:YFP for visualizing the anal depressor.

The plasmid containing the *aex-2* promoter (pBL348) (LeBoeuf and Garcia, 2017) was recombined with pGW77YFP to generate pBL351. 20 ng/µl of pBL351 was inject with 30 ng/µl of pLR361[Pges-1:RFP] and 150 ng/µl of pUC18 into N2. After a stable transgenic line was obtained, the extrachromosomal array was integrated into chromosome II to generate *rgIs21*.

CRISPR/Cas9-mediated recombination of YFP into genomic *pck-2* and *egl-2*

We used CRISPR/Cas9 to explore the expression of PCK-2, since we could not get reliable expression from DNA sequences upstream of the first *pck-2* ATG. Possibly, the *pck-2* promoter has expression elements in its large second intron. The CRISPR/Cas9 plasmids and protocols established in Dickinson *et al*. 2013 were used in this report (Dickinson et al., 2013).

To generate the genomic *pck-2*::YFP knock-in, a hundred ~ 5 to 10 hour old adult *unc-119(ed3)* hermaphrodites were microinjected with 50 ng/µl of the CRISPR/Cas9 plasmid pXG67 and 50 ng/µl the *pck-2* –YFP-*Cb unc-119* repair template pXG68. In the F2-F3 generation, non-paralyzed animals were

picked and analyzed by PCR and sequencing to verify homologous recombination between the repair template and the genomic *pck-2* locus. *Cb unc-119* was then excised from the animals by injecting germline expressing Cre recombinase from the plasmid pDD104 (Dickinson et al., 2013). The animals were then crossed with wild type (N2) to remove *unc-119(ed3)*. The *pck-2(rg551)*:YFP knock-in allele is referred to as *pck-2*::YFP.

To generate the genomic *egl-2*::YFP knock-in, a hundred ~ 5 to 10 hour old adult N2 hermaphrodites were microinjected with 50 ng/µl of the CRISPR/Cas9 plasmid pJG3 and 50 ng/µl the *egl-2*::YFP repair template pJG2. In the F2 generation, single animals were separated to generate independent lines. Samples from each line were screened, using PCR, to determine if they had YFP integrated in their genome. The strain *egl-2(rg444)*:YFP was screened positive for YFP and was verified that YFP was physically linked with the native *egl-2* loci on chromosome V. The K^+ channel:YFP fusion was found to be expressed on the cell surface of neurons and muscles and the cilia endings of sensory neurons.

To generate hypodermal marker, the *dpy-7* promoter from pXG76 was recombined upstream of DsRed in pGW322DsRed using LR clonase to generate pXG85.To distinguish individual muscle, injection mixes containing intestinal or hypodermal cell, 5 ng/µl muscle marker pLR132 (P_{lev-11}:DsRed) (Guo et al., 2012), 5 ng/µl hypodermal marker pXG85 and 190 ng/µl of pUC18 filler DNA, were injected into the germline of the *pck-2*::YFP strain.

CRISPR/Cas9-mediated mutation of *pck-2*::YFP

rgIs29[P_{pck-1}::TIMER] is integrated on chromosome I near *pck-2*. To generate animals that contain *rgIs29*[P_{pck-1}::TIMER] and a loss-of-function mutation in *pck-2*, we first constructed a *rgIs29*[P_{pck-1}::TIMER] *pck-2*::YFP recombinant. We then used the CRISPR/Cas9 plasmid pYW52, which is directed to the third *pck-2* exon, to generate an insertion/deletion mutation. We isolated *pck-2(rg800, rg551)* that disrupts *pck-2*::YFP expression; this allele is referred to as *pck-2(lf).* CRISPR/Cas9 generated a stop mutation by changing the wild-type sequence GGA GAG TAC TTC TGG GAG to GGA GAG TAA CGG GAG. We tested the copulation potency of the mutant *rgIs29*; *pck-2(lf)* as well as the heterozygote *pck-2*::YFP/*pck-2(lf)*; *rgIs29* to confirm the mutations and function of *pck-2*. As expected, mutant carrying *pck-2(lf)* showed similar accelerated decline as *pck-2(0)* (42% potent at 48 hr adulthood; n=50) while the heterozygote was as potent as wild type (84% potent at 48 hr adulthood; n=50).

CRISPR/Cas9-mediated mutation of *sdha-1*

To generate the loss-of-function mutation *rg448* in *sdha-1*, a hundred ~ 5 to 10 hour old adult hermaphrodites of the strain CG1367, which contain *pck-2*::*YFP (rg551); him-5(e1490)* were microinjected with 50 ng/µl of the CRISPR/Cas9 plasmid pXG128. In the F2-F3 generation, higher YFP-fluorescing L4 hermaphrodites were picked using a dissecting scope. The *sdha-1* region was sequenced and the CRISPR/Cas9 lesion changed the wild type sequence from GTC TGT GTC GGA GTC ATC GCC to the mutant sequence GTC TG**A GTC AG**T CGG AGT CAT CGC C.

Quantification of fluorescence

Digital images of animals were obtained using a Hamamatsu ImagEm camera mounted to an Olympus BX51 microscope (Olympus Corporation, Tokyo Japan) or a Yokogawa CSU-X1 Spinning Disk Unit (Andor Technology, CT USA) mounted on an Olympus IX81 microscope. Animals were anesthetized using abamectin (Sigma-Aldrich) (Cully et al., 1994). The more commonly used anesthetic NaN_3 was not used, since we found that paralyzing levels of the compound caused PCK-2::YFP to highly aggregate in the intestine, epidermis and muscles; the aggregation was reversible if the animals were removed from NaN_3. Acute paralytic levels of cyanide, rotenone and oligomycin also induced PCK-2::YFP aggregation; however, non-paralytic levels of these mitochondrial poisons caused much fewer aggregates to occur. PCK-1::YFP, expressed from transgenes, also aggregates under these conditions and when over-expressed. The aggregation, which confounded our data collection, might be related to the phenomenon where glycolytic enzymes accumulate at *C. elegans* synapses during energy stress (Jang et al., 2016). A 50 mg/ml DMSO stock of abamectin was diluted in M9 buffer to the working concentration of 250 µg/ml.

50 µl of the well-suspended colloidal abamectin solution was spotted on the surface of a 3 cm NGM plate containing OP50 bacteria. After the abamectin soaked into the agar, worms were then added to the abamectin-containing plate. Generally, 5 to 10 minutes elapsed before the worms became paralyzed. Independent animals were imaged at L1 through the 3rd day of adulthood. The adults were imaged using the 10X objective; L1 through L4 were imaged using a 20X objective.

Metamorph image software (version 7.8.0.0., Molecular Devices, Sunnyvale CA) was used to quantify average pixel intensity of a region of interest (ROI). To quantify $P_{pck\text{-}1}$:TIMER, a single line ROI was drawn along the whole ventral length of the animal. The ROI includes the ventral body wall muscle and ventral cord neurons. Average pixel intensity was determined for the line ROI. To quantify intestinal PCK-2::YFP expression in *pck-2(rg551)*; *pha-1(e2123); him-5(e1490)* males over-expressing PCK-1::YFP, a single line ROI was drawn along the animal's intestine. To quantify wild type PCK-2::YFP in different tissues, rectangular ROIs were drawn in accordance with the hypodermal and the muscle markers. Average pixel intensity was then determined for each ROI corresponding to PCK-2::YFP level in the cell. To quantify PCK-2::YFP expression in the whole animal, a series of rectangular ROIs were drawn that encompass the whole area of the worm. The ROI includes regions of the epidermis, pharynx, body wall muscles and intestine. Average pixel intensity was determined for each ROI and a single average of all ROIs was calculated for the whole worm. To quantify EGL-2::YFP, during microscopy data acquisition, a stack of 15 images was taken. ROIs were drawn over the protractor muscles or the SPD, SPC and SPV neurons and the average intensity was quantified.

Subcellular colocalization analysis

MATLAB (version R2017a, The Mathworks, Natick MA) was used to quantify the colocalization between mitochondria and PCK-2::YFP. Images containing cells expressing both mitochondrial CYN-1::CFP and PCK-2::YFP were selected from confocal image stacks. A polygon ROI was drawn over the cell to exclude other cells and background from interference. Then individual pixel intensity was quantified from the ROI for both CFP and YFP fluorescence. MATLAB was then used to generate the scattered plot of the pixels' intensities of the two channels, and calculate the Pearson's correlation coefficient (PCC) and R^2 (Dunn et al., 2011). As a positive control, wild type animals expressing two mitochondrial markers (mitoCFP and PDHA-1::YFP) were used with the same procedures performed.

Quantification of mitochondria size

MATLAB (version R2017a, The Mathworks, Natick MA) was used to quantify mitochondria size from confocal images. Images containing mitochondrial tagged CFP expressed in lateral epidermis were selected from confocal image stacks. To isolate the mitochondria signal from background noise, the pixels were filtered by cut off the intensity value with the local mean of 21 by 21 pixel region. Then, the grey-scale images were converted to black-white images with manually set thresholds so that the converted images were most consistent with the perceptions of the mitochondria signals. With an in-house script, connected white pixels reflecting mitochondria signals were quantified. Each cluster of connected white pixels represents individual mitochondrion, and the number of pixels were counted and converted to μm^2 to represent the sizes of the mitochondria. Clusters with less than 10 pixels each were treated as random noise and excluded from quantification.

ATP content quantification

The ATP quantification method was adapted from previous methods (Palikaras and Tavernarakis, 2016). For each sample, 20 developmentally synchronized worms were picked using Tris buffer (10 mM Tris-Cl, pH=8.0). The bacteria on the worms were cleaned off by washing the worms on an unseeded NGM plate with the Tris buffer. The worms were then transferred to a 0.2 mL PCR tube with 20 µL of the Tris buffer and ~20 0.5 mm Zirconium Oxide beads (Next Advance). For NaN_3 treated samples, the Tris buffer was replaced with 20 µL of Tris buffer containing 10 mM NaN_3. The worms were incubated in the NaN_3 containing Tris buffer for 10 minutes at 20°C before proceeding to the next step. The tube was then immersed and fast-frozen in the dry ice-ethanol bath for 5 minutes. After frozen, the tube was then heated to 95°C for 15 minutes in a PCR machine. To guarantee the release of ATP from all cells, tubes containing the Zirconium Oxide beads were put in a bullet blender (Next Advance) for 3 minutes at speed

setting level 8. The tubes were then spun down with a table top centrifuge and frozen at -80°C before the assay. On the day of the assay, the tubes were defrosted, and 180 µL Tris buffer was added into individual tubes to dilute the content by 10 fold. For ATP standards, 1 µM, 0.1 µM, or 0.01 µM ATP in the Tris buffer was used. The ATP content was determined using CellTiter-Glo Luminescent Cell Viability Assay Kit (Promega) with a Synergy HT plate reader (BioTek Instrument) following the manufacturer's instructions.

EMS mutagenesis

The *sdha-1(rg550)* allele, described in this study, was isolated from an ethyl methanesulfonate (EMS) screen for higher fluorescing *PCK-2*::YFP-containing worms. Worms were mutagenized following standard published methods (Anderson, 1995). ~5,000 hermaphrodites of the strain CG1367, which contain *pck-2*::*YFP; him-5(e1490)*, were mutagenized with EMS. In the F2 generation, higher YFP-fluorescing L4 hermaphrodites were picked using a dissecting scope. The *rg550* strain was outcrossed multiple times to the parental CG1367 strain to reduce background mutations. Whole genome sequencing was conducted by BGI Americas Corporation, in combination with further SNP mapping (Wicks et al., 2001), to locate the *rg550* allele within the *sdha-1* on chromosome X. The *rg550* mutation changed the wild type sequence GTC TGT GTC G**G**A GTC ATC GCC to the mutant sequence GTC TGT GTC G**A**A GTC ATC GCC (Gly to Glu).

Real-time PCR

The sequences for primers that anneal to *pyc-1, icl-1, pck-1,* and *pck-2* were described previously (Castelein et al., 2008). Three biological replicates of 500 day 1 adult males were accumulated over a period of time. Total RNA was extracted with TRI Reagent (Sigma-Aldrich) as previously described (Correa et al., 2012). cDNA was then synthesized with SuperScript II (Invitrogen) according to the protocol using around 2 µg total RNA and a mixture of gene specific-primers targeting the 20 genes. The mixture of gene specific-primers contains 5 pmole of reverse primers for each of the 20 genes. The RT-qPCR reactions were performed using BIO-RAD CFX96 real-time system and SsoFast EvaGreen supermix. We picked 10 reference genes (*act-1, ama-1, csq-1, tba-1, gpd-3, mdh-1, pat-10, mlc-3, mua-6* and *unc-15*) for the study. geNorm v3 (Vandesompele et al., 2002) was used to determine the most consistent reference gene from the 10 candidate reference genes. *unc-15* was selected as the reference gene to normalize the expression of the metabolic genes.

Paraquat assay

Prepared NGM plates were allowed to soak with Paraquat CL tetrahydrate (Supelco) at a final concentration of 10 mM. Plates were then spotted with concentrated OP50 and allowed to dry overnight. L4 males were then placed on plates and allowed to grow for 24-48 hours. To assess survival, worms were suspended in M9 buffer for 10 seconds and monitored for thrashing. If worms trashed more than once then they were considered alive. If one or no thrashing events were observed worms were considered incapacitated or dead.

Nile Red staining and quantification

The following was adapted from (Pino et al., 2013). Briefly, 15-20 L4 males were isolated from hermaphrodites and incubated on NGM plates the night before staining. The next day, males were transferred using 150 µL PBS with 0.01% triton X-100 (Sigma-Aldrich) to a 1.5 mL Eppendorf tube, centrifuged and the supernatant was aspirated leaving the worm pellet. Animals were then fixed using 150 µL of 40% isopropanol and incubated at room temperature for 3 min. During this incubation, 1 mL of Nile Red (Invitrogen™ Molecular Probes™) stain was prepared using 6 µL Nile Red stock solution (0.5 mg/ml in acetone) per 1 mL of 40% isopropanol. Males were pelleted by centrifugation and the supernatant was aspirated. To each worm pellet, 150 µL of Nile red staining solution was added and males were stained in the dark for 2 hours. Males were then collected by centrifugation and supernatant was aspirated. Males were washed with 150 µL of PBS with 0.01% triton X-100 in the dark for 30

minutes. Males were centrifuged and carefully transferred to a glass slide with 2% noble agar pad before imaging.

Males were imaged on a Yokogawa CSU-X1 Spinning Disk Unit mounted on an Olympus IX81 microscope (514 nm laser) using a 10X objective. Metamorph imaging software was used to quantify the average intensity of intestinal neutral lipid stores. The ROI was drawn exclusively in the intestine using a series of 10 by 10 pixel rectangular ROI's for males. Each rectangular ROI was used to calculate the average intensity in a segment of the intestine. Specific segments could then be averaged for each worm. Nile Red staining was occluded by hermaphrodite eggs therefore we treated hermaphrodites and males similarly by only calculating average intensity using the first five segmented ROI's.

Oxygen consumption assay

Oxygen consumption was measured using lab-made respirometers (Zhang et al., 2015). A chunk of NGM agar, containing a 5 mm lawn of UV-killed OP50 and 300 day 1 wild type or *sdha-1(rg550)* hermaphrodites, was assembled into the respirometer. The respirometer contained soda lime (Sigma-Aldrich), which absorbs expelled CO_2 and thus decreases the pressure within the respirometer. The change in gas volume (decreased pressure) is measured by the rise of colored fluid in a glass capillary attached to the closed respirometer. The respirometers were kept in a constant temperature room (20°C). The assembled respirometer with the worms were allowed to adjust (balance) for 20 min before measurements were taken. The respirometers were not handled during the experiment; data were collected by taking digital photographs of the rising liquid in the capillary tube.

Measuring male body bends in M9 buffer

In groups of 10 animals, L4 males (either wild type or *sdha-1(rg550)*) from non-crowded cultures were separated from their hermaphrodite siblings. The next day, groups of 5 males were transferred to a 3-well glass Pyrex dish containing 1 ml of M9 salts buffer. Males were digitally recorded using an Olympus SZX16 stereomicroscope fitted with a Hamamatsu C4742-95 digital camera. Images were taken at a rate of 30 frames a second for 1 minute. On playback, the recordings were slowed down five to ten times to count the body bends, as the males thrashed in the M9 buffer.

Egg-Laying assays

To examine the effect of *sdha-1* mutation on egg-laying behavior, L4 hermaphrodites were isolated 24 hours before the assay. For the assay, worms were individually transferred to new NGM plates seeded with OP50 and allowed to lay eggs for two hours. Number of eggs was then counted.

Glycogen staining

To stain glycogen, 1-day-old virgin males were stained in 100 µL of diluted (1:10) Lugo's iodine solution (2% I_2 in 4% KI) in dark for 2 min (Frazier HN and MB., 2009). The worms were then transferred to microscope slides. 16-bit grey scale images of the isthmus region of pharynx were taken with Olympus BX51 compound microscope mounted with a Hamamatsu ImagEm camera and 520/30 nm light filter. The brown-red light of glycogen staining was absorbed by the light filter and darker in the image. ImageJ (Schneider CA et al., 2012) was used to quantify the mean grey level of the isthmus regions and the background. Relative absorbance of the glycogen staining was calculated using the formula: $A = \frac{Tb-Ti}{Tb}$, where Tb is the mean grey level of the background and Ti is the mean grey level of the isthmus region.

Growth of *C. elegans* in mitochondrial poisons

All reagents and plates were prepared freshly. Oligomycin (Sigma-Aldrich) and rotenone (Sigma-Aldrich) were dissolved in DMSO to make a stock concentration of 10 mg/ml and 100 mM, respectively. Sodium azide (EM Science) was dissolved in water to make a stock concentration of 100 mM. Stock solutions were diluted in M9 and added separately to NGM plates containing OP50 to make the final concentration 10 µg/ml of oligomycin, 10 µM of rotenone and 15 µM of sodium azide. For controls, the same volume of DMSO was diluted in M9 and then added to OP50-seeded NGM plates. Each concentration of

mitochondrial inhibitor was selected to induce slow developmental growth with ~10% lethality. 10 L4 *pck-2*:YFP were put onto each drug or control plate. In the next generation, ~40 L4 progeny were transferred to freshly prepared drug plates to be imaged. L4 worms were imaged on the same day, day 1 adult worms were imaged after 24 hrs, day 2 worms after 48 hrs and day 3 worms after 72 hrs. YFP fluorescent images were taken using a Hamamatsu ImageEm digital camera on an Olympus BX51 at 10X magnification.

For malonate treatment, plates were prepared similar to NGM plates, but without peptone. Additionally, OP50 was killed by UV exposure to prevent the bacteria from metabolizing malonate. Sodium malonate dibasic was dissolved in sterile water to make a stock concentration of 2 M. For the control, 4 ml of peptone-less agar media was poured into a 3.5 mm petri dish. To prepare the 50 mM malonate plates, 100 µl of 2M sodium malonate (Bean Town Chemicals) was mixed into of 4 ml molten peptone-less agar media and then poured into a 3.5 mm petri dish. After the agar solidified, UV-killed OP50 was added to the plates. Unlike the mitochondrial inhibitors, the malonate quickly lost its toxicity, thus ~30 L3 wildtype or *sdha-1(rg550)I* hermaphrodites were placed on each plate and after 24 and 72 hrs, YFP fluorescent images were taken.

To quantify PCK-2::YFP expression in the whole animal, a series of rectangular ROIs were drawn that encompass the whole area of the worm. The ROI includes regions of the epidermis, pharynx, body wall muscles and intestine. Average pixel intensity was determined for each ROI and a single average of all ROIs was calculated for the whole worm.

Supplemental References

Anderson, P. (1995). Mutagenesis. In *Caenorhabditis elegans*: Modern Biological Analysis of an Organism, H.E. Epstein, and D.C. Shakes, eds. (San Diego, CA: Academic Press), pp. 31-58.

Aroian, R.V., and Sternberg, P.W. (1991). Multiple functions of *let-23*, a *Caenorhabditis elegans* receptor tyrosine kinase gene required for vulval induction. Genetics *128*, 251-267.

Banerjee, S., Versaw, W.K., and Garcia, L.R. (2015). Imaging cellular inorganic phosphate in *Caenorhabditis elegans* using a genetically encoded FRET-based biosensor. PLOS ONE *10*, e0141128.

Brenner, S. (1974). The genetics of *Caenorhabditis elegans*. Genetics *77*, 71-94.

Correa, P., LeBoeuf, B., and Garcia, L.R. (2012). *C. elegans* dopaminergic D2-like receptors delimit recurrent cholinergic-mediated motor programs during a goal-oriented behavior. PLOS Genet *8*, e1003015.

Cully, D.F., Vassilatis, D.K., Liu, K.K., Paress, P.S., Van der Ploeg, L.H.T., Schaeffer, J.M., and Arena, J.P. (1994). Cloning of an avermectin-sensitive glutamate-gated chloride channel from *Caenorhabditis elegans*. Nature *371*, 707-711.

Dickinson, D.J., Ward, J.D., Reiner, D.J., and Goldstein, B. (2013). Engineering the Caenorhabditis elegans genome using Cas9-triggered homologous recombination. Nat Methods *10*, 1028-1034.

Dunn, K.W., Kamocka, M.M., and McDonald, J.H. (2011). A practical guide to evaluating colocalization in biological microscopy. Am J Physiol Cell Physiol *300*, C723-742.

Frazier HN, and MB., R. (2009). Adaptive sugar provisioning controls survival of c. elegans embryos in adverse environments. Curr Biol *19*, 859-863.

Gilleard, J., Barry, D., and Johnstone, I. (1997). Cis regulatory requirements for hypodermal cell-specific expression of the *Caenorhabditis elegans* cuticle collagen gene *dpy-7*. Mol Cell Biol *17*, 2301-2311.

Granato, M., Schnabel, H., and Schnabel, R. (1994). *pha-1*, a selectable marker for gene transfer in *C. elegans*. Nucleic Acids Res *22*, 1762-1763.

Hodgkin, J., and Doniach, T. (1997). Natural variation and copulatory plug formation in *Caenorhabditis elegans*. Genetics *146*, 149-164.

Hodgkin, J.A., Horvitz, H.R., and Brenner, S. (1979). Nondisjunction mutants of the nematode *Caenorhabditis elegans*. Genetics *91*, 67-94.

Jang, S., Nelson, Jessica C., Bend, Eric G., Rodríguez-Laureano, L., Tueros, Felipe G., Cartagenova, L., Underwood, K., Jorgensen, Erik M., and Colón-Ramos, Daniel A. (2016). Glycolytic enzymes localize to synapses under energy stress to support synaptic function. Neuron *90*, 278-291.
Kimble, J., and Hirsch, D. (1979). The post embryonic cell lineages of the hermaphrodite and male gonads in *Caenorhabditis elegans.* Dev Biol *70*, 396-417.
LeBoeuf, B., and Garcia, L.R. (2017). Caenorhabditis elegans Male Copulation Circuitry Incorporates Sex-Shared Defecation Components To Promote Intromission and Sperm Transfer. G3 (Bethesda) *7*, 647-662.
Newbury, R.H., Viveiros, R.D., Johnsen, R.C., Mah, A.K., Anastas, D., Fang, L.L., Halfnight, E., Lee, D., Lin, J., Lorch, A.H.*, et al.* (2007). High-throughput *in vivo* analysis of gene expression in *Caenorhabditis elegans.* PLOS Biol *5*, e237.
Palikaras, K., and Tavernarakis, N. (2016). Intracellular Assessment of ATP Levels in Caenorhabditis elegans. Bio Protoc *6*.
Pino, E.C., Webster, C.M., Carr, C.E., and Soukas, A.A. (2013). Biochemical and high throughput microscopic assessment of fat mass in *Caenorhabditis elegans*. J Vis Exp, 50180.
Schedl, T., and Kimble, J. (1988). *fog-2*, a germ-line-specific sex determination gene required for hermaphrodite spermatogenesis in *Caenorhabditis elegans*. Genetics *119*, 43-61.
Schnabel, H., and Schnabel, R. (1990). An organ-specific differentiation gene, *pha-1*, from *Caenorhabditis elegans*. Science *250*, 686-688.
Schneider CA, Rasband WS, and KW., E. (2012). NIH Image to ImageJ: 25 years of image analysis. . Nat Methods *9*, 671-675.
Teramoto, T., Lambie, E., and Iwasaki, K. (2005). Differential regulation of TRPM channels governs electrolyte homeostasis in the *C. elegans* intestine. Cell Metab *1*, 343-354.
Vandesompele, J., De Preter, K., Pattyn, F., Poppe, B., Van Roy, N., De Paepe, A., and Speleman, F. (2002). Accurate normalization of real-time quantitative RT-PCR data by geometric averaging of multiple internal control genes. Genome Biol *3*, research0034.0031.
Wicks, S.R., Yeh, R.T., Gish, W.R., Waterston, R.H., and Plasterk, R.H. (2001). Rapid gene mapping in *Caenorhabditis elegans* using a high density polymorphism map. Nat Genet *28*, 160-164.
Zhang, L., Gualberto, D.G., Guo, X., Correa, P., Jee, C., and Garcia, L.R. (2015). TMC-1 attenuates *C. elegans* development and sexual behaviour in a chemically defined food environment. Nat Commun *6*, 6345.
